# On the origin and evolution of RNA editing in metazoans

**DOI:** 10.1016/j.celrep.2023.112112

**Published:** 2023-02-14

**Authors:** Pei Zhang, Yuanzhen Zhu, Qunfei Guo, Ji Li, Xiaoyu Zhan, Hao Yu, Nianxia Xie, Huishuang Tan, Nina Lundholm, Lydia Garcia-Cuetos, Michael D. Martin, Meritxell Antó Subirats, Yi-Hsien Su, Iñaki Ruiz-Trillo, Mark Q. Martindale, Jr-Kai Yu, M. Thomas P. Gilbert, Guojie Zhang, Qiye Li

**Affiliations:** 1 BGI-Shenzhen, Shenzhen 518083, China; 2 Section for Ecology and Evolution, Department of Biology, University of Copenhagen, 2100 Copenhagen, Denmark; 3 BGI Research-Wuhan, BGI, Wuhan 430074, China; 4 College of Life Sciences, University of Chinese Academy of Sciences, Beijing 100049, China; 5 Natural History Museum of Denmark, University of Copenhagen, 1353 Copenhagen, Denmark; 6 Department of Natural History, NTNU University Museum, Norwegian University of Science and Technology (NTNU), 7491 Trondheim, Norway; 7 Center for Theoretical Evolutionary Genomics, Department of Integrative Biology, University of California Berkeley, Berkeley, CA 94720, USA; 8 Institute of Evolutionary Biology, UPF-CSIC Barcelona, 08003 Barcelona, Spain; 9 Institute of Cellular and Organismic Biology, Academia Sinica, Taipei 11529, Taiwan; 10 ICREA, Passeig Lluís Companys 23, 08010 Barcelona, Catalonia, Spain; 11 Departament de Genètica, Microbiologia i Estadística, Facultat de Bilogia, Universitat de Barcelona (UB), 08028 Barcelona, Spain; 12 The Whitney Laboratory for Marine Bioscience, University of Florida, St. Augustine, FL 32080, USA; 13 Marine Research Station, Institute of Cellular and Organismic Biology, Academia Sinica, Yilan 26242, Taiwan; 14 Center for Evolutionary Hologenomics, The GLOBE Institute, University of Copenhagen, 1353 Copenhagen, Denmark; 15 Center of Evolutionary and Organismal Biology, & Women’s Hospital, School of Medicine, Zhejiang University, Hangzhou, China; 16 Liangzhu Laboratory, Zhejiang University Medical Center, 1369 West Wenyi Road, Hangzhou 311121, China; 17 State Key Laboratory of Genetic Resources and Evolution, Kunming Institute of Zoology, Chinese Academy of Sciences, Kunming 650223, China; 18 Villum Centre for Biodiversity Genomics, Section for Ecology and Evolution, Department of Biology, University of Copenhagen, 2100 Copenhagen, Denmark

**Keywords:** Holozoa, animal, RNA editing, Adar, A-to-I editing, evolution, sense-antisense, recoding editing, neural system, cytoskeleton

## Abstract

Extensive adenosine-to-inosine (A-to-I) editing of nuclear-transcribed mRNAs is the hallmark of metazoan transcriptional regulation. Here, by profiling the RNA editomes of 22 species that cover major groups of Holozoa, we provide substantial evidence supporting A-to-I mRNA editing as a regulatory innovation originating in the last common ancestor of extant metazoans. This ancient biochemistry process is preserved in most extant metazoan phyla and primarily targets endogenous double-stranded RNA (dsRNA) formed by evolutionarily young repeats. We also find intermolecular pairing of sense-antisense transcripts as an important mechanism for forming dsRNA substrates for A-to-I editing in some but not all lineages. Likewise, recoding editing is rarely shared across lineages but preferentially targets genes involved in neural and cytoskeleton systems in bilaterians. We conclude that metazoan A-to-I editing might first emerge as a safeguard mechanism against repeat-derived dsRNA and was later co-opted into diverse biological processes due to its mutagenic nature.

## Highlights

• A-to-I editing is a regulatory innovation originating in the ancestor of metazoans
• Evolutionarily young repetitive elements are the primary targets of A-to-I editing
• Editing on pairing regions of sense-antisense transcripts is common in some species
• Recoding editing convergently targets neural and cytoskeleton systems in Bilateria

## Graphical abstract

**Figure:**
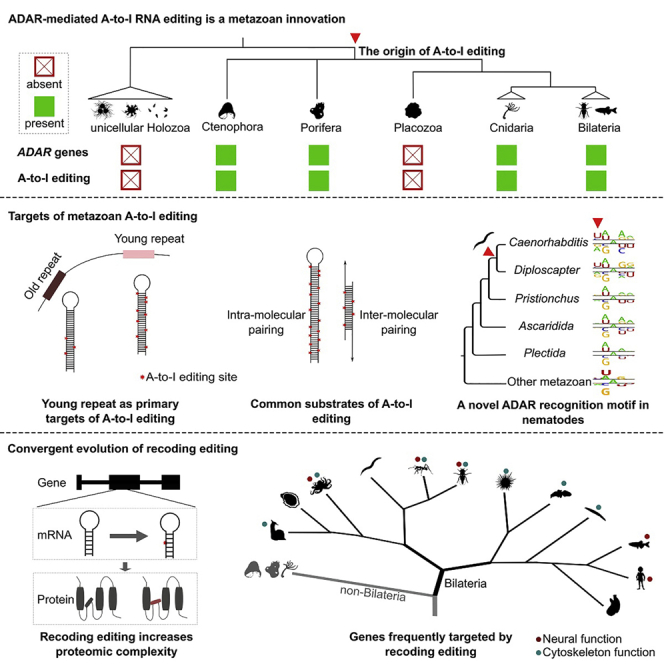

## Teaser

Zhang et al. profile the RNA editomes of 22 species that cover major groups of Holozoa. Their findings support ADAR-mediated A-to-I editing as a metazoan innovation, which might have first emerged as a safeguard mechanism against repeat-derived dsRNA and was later co-opted into other biological processes due to its mutagenic nature.

## Introduction

The central dogma of molecular biology emphasizes how genetic information passes faithfully from DNA to RNA to proteins.^1^ However, this dogma has been challenged by the phenomenon of RNA editing, which creates RNA products that differ from their DNA templates.^2^ RNA editing systems have arisen multiple times within eukaryotes and involve a range of posttranscriptional processing mechanisms that alter RNA sequences by the insertion, deletion, or substitution of nucleotides but exclude splicing, 5′-capping, and 3′-polyadenylation by convention.^2^^,^^3^ In metazoans, adenosine (A)-to-inosine (I) editing catalyzed by double-stranded RNA (dsRNA)-specific adenosine deaminases (ADARs) is the most abundant form of RNA editing.^4^^,^^5^ This ADAR-mediated editing system is remarkable among all editing systems discovered in eukaryotes thus far, as it can modify a large set of nuclear-transcribed mRNAs, while editing in other eukaryotes mainly targets a handful of tRNAs and organellar mRNAs.^2^

As inosine is interpreted by ribosomes and other molecular machineries as guanosine and base pairs with cytosine, A-to-I editing can result in alterations to the coding potential or structural properties of mature RNAs.^4^^,^^5^^,^^6^ A-to-I editing has been demonstrated to occur in diverse organs and tissues in model metazoans,^7^^,^^8^^,^^9^^,^^10^ in which it has been shown to modulate developmental processes,^8^^,^^11^^,^^12^^,^^13^ neural network plasticity,^14^^,^^15^ immune responses,^16^^,^^17^ skeletal myogenesis,^18^ hematopoiesis,^19^ and organismal adaptation to environmental changes.^20^^,^^21^^,^^22^ Defects in the editing machinery have been linked to neurological diseases, autoimmune disorders, and even cancers in humans.^23^^,^^24^^,^^25^

However, while we are marveling at the versatility of ADAR-mediated A-to-I editing, with new biological roles still being discovered, our knowledge about the origin, evolutionary dynamics, and general role of this mechanism in the animal kingdom is limited. For example, A-to-I editing is found to occur widely in eumetazoans from coral to humans,^4^ but whether this regulatory mechanism had already emerged in earlier-branching metazoans or even their closest unicellular relatives remains unknown. The majority of A-to-I editing events in many studied metazoans reside in repetitive elements,^26^^,^^27^ suggesting a role in suppressing retrotransposon activity.^28^ However, there are also well-known exceptions, such as A-to-I editing primarily targeting exonic (particularly coding) regions in *Drosophila*.^29^^,^^30^ While recoding editing, which leads to nonsynonymous substitutions in protein-coding sequences, is abundant and affects almost half of the protein-coding genes in coleoids,^31^^,^^32^^,^^33^ it has been demonstrated to be quite rare in other examined animals.^13^^,^^34^^,^^35^^,^^36^^,^^37^^,^^38^ In addition, many of the metazoan A-to-I editing sites tend to appear in clusters due to hyper-editing of long dsRNA substrates, while functionally important recoding sites mainly appear as isolated sites due to site-selective editing.^39^ Therefore, from a technical perspective, systematic profiling of all RNA-editing sites in an organism is still challenging,^40^ which hinders the investigation of RNA editing at a broad phylogenetic scale.

Here, we leveraged a matching DNA and RNA sequencing strategy with an optimized RES-Scanner framework^41^ to profile the RNA editomes of representative species across the phylogeny of Holozoa, the clade that includes all extant animals and their closest single-celled relatives.^42^ Our comprehensive investigation into RNA editing from a phylogenetic perspective sheds light on the biological role and evolutionary principle of this posttranscriptional regulatory mechanism in the animal kingdom.

## Results

### Profiling of RNA editomes across the phylogeny of Holozoa

**Figure 1:**
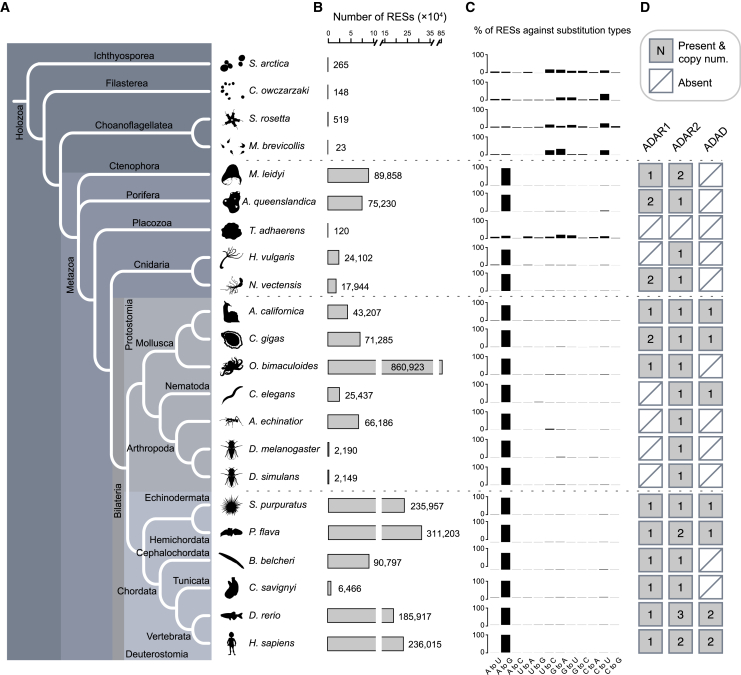
The distribution of *ADAR/ADAD* genes and A-to-I mRNA editing in metazoans (A) The phylogeny of the 22 species examined in this study. The topology of the phylogenetic tree was derived according to previous reports.^44^^,^^45^^,^^46^ Full names for the 22 species from top to bottom are *Sphaeroforma arctica* (ichthyosporean); *Capsaspora owczarzaki* (filasterean); *Salpingoeca rosetta* (choanoflagellate); *Monosiga brevicollis* (choanoflagellate); *Mnemiopsis leidyi* (ctenophore); *Amphimedon queenslandica* (sponge); *Trichoplax adhaerens* (placozoan); *Hydra vulgaris* (hydra); *Nematostella vectensis* (sea anemone); *Aplysia californica* (sea hare); *Crassostrea gigas* (oyster); *Octopus bimaculoides* (octopus); *Caenorhabditis elegans* (roundworm); *Acromyrmex echinatior* (ant); *Drosophila melanogaster* (fruit fly); *Drosophila simulans* (fruit fly); *Strongylocentrotus purpuratus* (sea urchin); *Ptychodera flava* (acorn worm); *Branchiostoma belcheri* (lancelet); *Ciona savignyi* (sea squirt); *Danio rerio* (zebrafish); and *Homo sapiens* (human). (B) The total number of potential RNA-editing sites (RESs) identified in each species. (C) The percentage of editing sites across the 12 possible types of nucleotide substitutions. (D) The presence/absence of *ADAR1*, *ADAR2*, and *ADAD* in each metazoan species. The copy number is also indicated if a gene is present. See also Figures S1 and S2 and Tables S1 and S2.

We performed whole-genome DNA sequencing (DNA-seq) and strand-specific RNA-seq on 18 species, including 14 metazoans and four unicellular eukaryotes closely related to animals. For each species, two to three (mostly three) specimens were sequenced to serve as biological replicates, with the average DNA and RNA coverage achieving 75× and 45×, respectively, for each specimen after sequence alignment (Table S1). Together with the published sequencing data from the nematode *Caenorhabditis elegans*,^13^ the ant *Acromyrmex echinatior*,^34^ the octopus *Octopus bimaculoides*,^43^ and humans,^10^ we were able to profile and compare the RNA editomes of 22 species that represented nearly all the major phyla of extant metazoans as well as their closest unicellular relatives (Figure 1A).

Two complementary methods were adopted to identify the RNA-editing sites for each species. Briefly, we first employed RES-Scanner^41^ to identify editing sites by comparing the matching DNA- and RNA-seq data from the same specimen. This method has high accuracy when searching for RNA-editing sites that are isolated or not heavily clustered.^41^ We next performed hyper-editing detection following the approach originally proposed by Porath et al.^47^ and used the RNA reads that failed to align by RES-Scanner to capture the hyper-edited reads and the clusters of editing sites they harbored. The results of these two methods were finally combined to yield the RNA editome of each specimen (Table S1; see STAR Methods for details).

### A-to-I mRNA editing emerged as a regulatory innovation in the last common ancestor of modern metazoans accompanied by the origin of *ADAR*

We detected very few putative RNA-editing sites (ranging from 23–519) in the four unicellular relatives of metazoans (Figure 1B; Table S1). No dominant type of nucleotide substitution was observed (Figure 1C), and the frequency of each type of nucleotide substitution was close to that of genetic polymorphism (Figure S1A), implying that the RNA-editing sites detected in these species represented noise. In contrast, thousands to hundreds of thousands of potential RNA-editing sites were identified in almost all the examined metazoans, with the vast majority (>90%) consisting of A-to-G substitutions. The only exception was *Trichoplax adhaerens*, a morphologically simple metazoan from Placozoa.^48^

A-to-G substitutions in metazoan mRNA putatively result from ADAR-mediated A-to-I editing. Therefore, we next conducted a comprehensive search of the *ADAR* homologs in the genomes and transcriptomes of the 22 species and classified these homologs into *ADAR1*, *ADAR2*, or the catalytically inactivated *ADAD* based on protein phylogenetic analyses. We found that *ADARs* exist in all investigated species except *T. adhaerens* and the unicellular taxa, and more importantly, most metazoans, including the ctenophore and the sponge, have orthologs of human *ADAR1* and *ADAR2* (Figures 1D and S2; Table S2). This indicates that at least one *ADAR1* and one *ADAR2* existed in the last common ancestor (LCA) of extant metazoans. Second, our phylogenetic analysis placed the previously unclassified orphan *ADAR* of *Hydra vulgaris* in the *ADAR2* clade, suggesting that this model cnidarian underwent a secondary loss of the ancestral *ADAR1* during evolution, a scenario that is also observed in insects^49^^,^^50^ (Figures 1D and S2). *ADAD*s were only identified in some protostomes and deuterostomes but are sister groups of all metazoan *ADAR1*s (Figures 1D and S2), implying that the first *ADAD* might also emerge in the LCA of extant metazoans.

Nevertheless, regardless of copy-number variation between species, we found that the existence of *ADAR* genes across the metazoan phylogenetic tree fit perfectly with the existence of extensive A-to-G substitutions in their transcriptomes (Figure 1). Our results thus strongly support that ADAR-mediated editing of nuclear-transcribed mRNAs is a posttranscriptional regulatory mechanism originating in the LCA of modern metazoans. We also highlight that our detection method does not depend on any prior knowledge about the dominant type of RNA editing in any species; thus, our results also imply that RNA editing in any manner other than A-to-I is either extremely rare or nonexistent in the animal kingdom (Figure S1B). This prompted us to focus only on A-to-I editing in all downstream analyses.

### Evolutionarily young repetitive elements are the primary targets of metazoan A-to-I editing

We next compared the genomic targets of A-to-I editing in a broad phylogenetic context. To overcome the potential biases caused by the variable accuracy of repeat annotation in different species, we reannotated the repetitive elements of all investigated species in the first step (see STAR Methods). To confirm the reliability of our method, we compared our annotation results of the fruit fly *Drosophila melanogaster* and the zebrafish *Danio rerio* with those downloaded from UCSC and observed very good consistency (Figures S3A and S3B).

**Figure 2:**
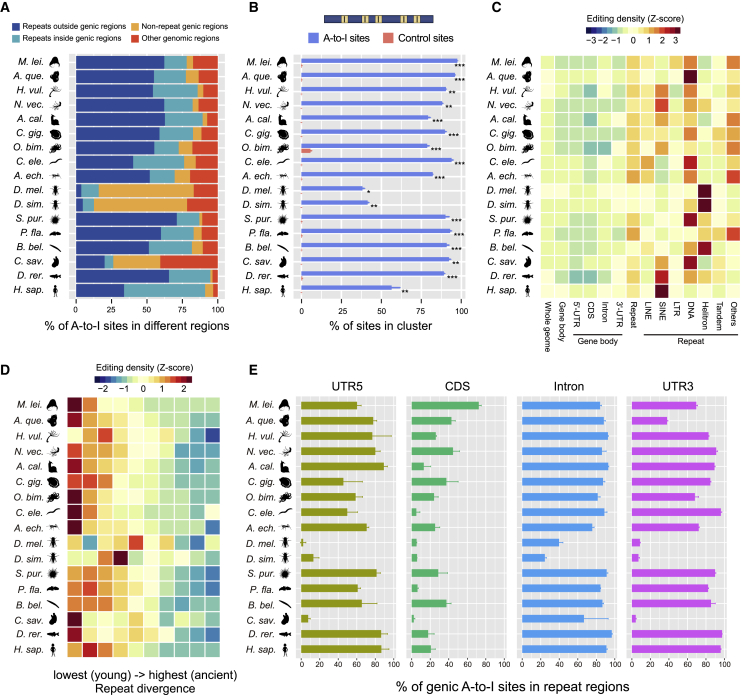
The genomic targets of metazoan A-to-I editing (A) The proportion of A-to-I editing sites in different genomic regions. Genic regions include untranslated (5′ UTR and 3′ UTR), CDS, and intron regions of all protein-coding genes. Repeats include transposons and tandem repeats annotated for each species in this study. (B) The percentage of A-to-I editing sites occurring in clusters. A cluster contains ≥3 A-to-I editing sites, of which the distance between two adjacent sites is ≤30 nt. Control sites are randomly selected transcribed adenosines with the same number and comparable RNA depth of the A-to-I editing sites in each sample from each species. Bars represent the mean ± SD across samples, and asterisks indicate significance levels estimated by two-tailed paired t tests, with ^∗^p < 0.05, ^∗∗^p < 0.01, and ^∗∗∗^p < 0.001. (C) Comparison of editability across different genomic elements in each species. Editability is measured as the number of A-to-I editing sites per million transcribed adenosine sites (RNA depth ≥2×) for each type of genomic element. (D) The negative correlation between the sequence divergence and the editability of repetitive elements. (E) The percentages of genic A-to-I editing sites located in regions annotated as concurrent repetitive elements. Genic editing sites were defined as editing sites located in the 5′ UTR, CDS, intron, and 3′ UTR of protein-coding genes. Bars represent the mean ± SD across samples. See also Figure S3.

We found that in almost all investigated metazoans, including the earliest branching lineages (ctenophore and sponge), repetitive elements were unambiguously the primary targets of A-to-I editing and harbored on average 83% of the identified editing sites (Figure 2A). Of note, we also observed a high proportion of repeat-targeting editing sites in the sea hare *Aplysia californica* (87%) and the sea urchin *Strongylocentrotus purpuratus* (86%), in sharp contrast with the low percentages (7% in sea hare; 31% in sea urchin) reported previously, which were probably due to inaccurate repeat annotation.^26^ With regard to locational distribution, on average, 82% (ranging from 38% to 97%) of the A-to-I sites were estimated to be organized in clusters (Figure 2B), in agreement with the notion that most metazoan editing events resulted from the hyper-editing of long dsRNA substrates rather than site-selective editing.^4^^,^^39^ These results together suggest that hyper-editing of repeat-derived dsRNA is probably an ancient phenomenon that already occurred in the LCA of extant metazoans.

We further compared the editability of different genomic elements by counting the number of editing sites per million transcribed adenosine sites (i.e., the density of editable sites) for each type of genomic element. This revealed that the editability of genic elements (i.e., 5′ UTR, coding sequence [CDS], intron, and 3′ UTR) was close to the whole-genome average level in all metazoans. In contrast, repetitive elements, especially DNA transposons, short interspersed nuclear elements (SINEs), and long interspersed nuclear elements (LINEs), usually showed the highest editability (Figures 2C and S3C). Moreover, we observed negative correlations between the sequence divergence rate and the editability of repetitive elements (Figures 2D and S3D), indicating that A-to-I editing preferentially targets evolutionarily young repetitive elements that likely only relatively recently invaded the genome of each species. Considering that many editing sites on protein-coding genes were also located within repetitive elements (Figure 2E), we speculate that most editable positions on genes were originally introduced by the invasion of repetitive elements into genic regions during genome evolution.

However, the two *Drosophila* flies and the sea squirt *Ciona savignyi* represent rare exceptions regarding the primary genomic targets of A-to-I editing, with only 13%–24% of editing sites residing in repetitive elements (Figure 2A). By examining the repeatomes of all species, we found that the two *Drosophila* genomes are relatively devoid of repeats (Figure S3E). In addition, the *Drosophila* repeats showed the lowest probability of finding a nearby inverted copy relative to those in other metazoans (Figure S3F). These features together would likely reduce the number of dsRNA substrates formed by the pairing of two nearby repeats, which in turn would reduce repeat-derived editing sites in *Drosophila*. However, the *Drosophila* mechanism does not work for *C. savignyi* because up to 37% (66/177 Mb) of the *C. savignyi* genome sequences were annotated as repeats, a ratio comparable to many other metazoan genomes (Figure S3E), and the *C. savignyi* repeats displayed a moderate probability of finding a nearby inverted copy when compared with other metazoans (Figure S3F). However, we found that the transcriptional activity of the *C. savignyi* repetitive elements was extremely low, with only 1.7 Mb (2.6%) of repeat sequences achieving ≥2× RNA coverage (Figure S3G). This implies that depressed transcriptional activity rather than sequence degeneration in the *C. savignyi* repeatome leads to the reduction in repeat-derived editing sites in *C. savignyi*. Overall, these rare evolutionary exceptions provide valuable evidence supporting the notion that the property of the repeatome is critical for establishing the global RNA editome of a metazoan species.^26^^,^^27^

### Intermolecular pairing of sense and antisense transcripts is a neglected but important mechanism for forming dsRNA substrates for A-to-I editing

Although both *C. savignyi* and *Drosophila* editing sites showed similarly a low repeat-targeting preference (Figure 2A), there was also a notable difference. In contrast to the low proportion of clustered editing sites identified in *Drosophila*, we found that up to 92% of editing sites in *C. savignyi* appeared in clusters, a ratio comparable to that in other metazoans (Figure 2B). This feature suggested that most editing events in *C. savignyi* also resulted from the hyper-editing of long dsRNA substrates, as expected in many other metazoans. Then, a critical question arises: where did the long dsRNA substrates in *C. savignyi* come from if they were not from the conventional pairing of nearby inverted repeats?

**Figure 3:**
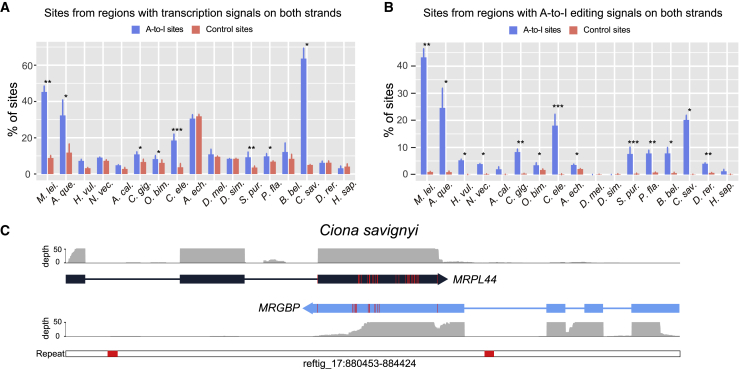
A-to-I editing of dsRNA substrates formed by intermolecular pairing of sense and antisense transcripts (A) The percentage of A-to-I editing sites located in dsRNA regions potentially formed by intermolecular pairing of sense-antisense transcripts and measured as the proportion of sites located in a region (±50 nt surrounding the focal edited adenosine) with a transcription signal (RNA depth ≥2× along >50% of the region) in both strands. Control sites are randomly selected transcribed adenosines with the same number and comparable RNA depth of the A-to-I editing sites in each sample of each species. (B) The proportion of A-to-I editing sites located in regions with editing signals on both strands and measured as the proportion of sites located in a region (±25 nt surrounding the focal edited adenosine) with at least one A-to-I editing site found on the opposite strand. The control sites are the same as those in (A). (C) An example of sense-antisense transcript pairing in *Ciona savignyi* showing the RNA coverage of both transcript models, the location of A-to-I editing sites on both transcripts (red vertical bars within each transcript model), and the distribution of repeats in this genomic region (red boxes in the bottom track). In (A) and (B), bars represent the mean ± SD across samples, and asterisks indicate significance levels estimated by two-tailed paired t tests, with ^∗^p < 0.05, ^∗∗^p < 0.01, and ^∗∗∗^p < 0.001. See also Table S3.

Intermolecular pairing of sense and antisense transcripts is another potential mechanism to form long dsRNAs,^51^ but its contribution to global A-to-I editing was considered to be negligible in humans and mice.^52^^,^^53^ Taking advantage of the strand information provided by strand-specific RNA-seq, we were able to reexamine the role of this mechanism in inducing A-to-I editing in diverse metazoans. By investigating the transcription signals in the opposite strand of each editing site and using randomly selected transcribed adenosines as control sites, we found that eight out of the 17 surveyed metazoans had significantly higher proportions of editing sites residing in regions with antisense transcription signals than in control sites, including the ctenophore (45%), the sponge (32%), *C. elegans* (18%), and, particularly, the sea squirt (64%; Figure 3A). However, we could not find a difference between editing sites and control sites in humans, in agreement with previous findings.^52^^,^^53^

ADAR enzymes usually edit adenosines on both strands of dsRNA substrates.^54^ Thus, if dsRNAs formed by pairing of sense and antisense transcripts are edited by ADARs, one would also expect to observe A-to-I editing on the pairing region of both transcripts. In line with this prediction, we identified a significantly higher-than-expected fraction of editing sites located in regions (51 nt surrounding the focal editing site) containing one or more editing sites in the opposite strand in many species. As a control, a set of randomly selected transcribed adenosines was almost impossible to find edited adenosines in the opposite strand (Figure 3B). Even so, one may still argue that finding A-to-I editing on both strands does not necessarily mean that the editing events were induced by sense-antisense pairing, as these events could be due to intramolecular pairing of the sense and antisense transcripts, respectively. We therefore conducted further filtering steps to completely rule out this possibility. Briefly, if a candidate site resided in a repeat region or in a region that showed reverse-complement alignment within its upstream or downstream sequence by BLAST search, we regarded this site to be an editing site derived from intramolecular pairing and removed it from the sense-antisense candidates (see STAR Methods for details). Nevertheless, even after such rigorous filtering, we still observed that some species had notable fractions of editing sites that could only be explained by sense-antisense pairing, such as the ctenophore (∼7.2%) and the sea squirt (∼13.9%; Table S3). Moreover, in half of the 17 metazoans examined, we found several to dozens of regions that were edited on both strands across biological replicates (Table S3). Representative examples included the *MRPL44* and *MRGBP* genes that were paired and extensively edited in their 3′ UTRs in *C. savignyi* (Figure 3C). However, sense-antisense gene pairs that were edited across species were not identified.

### A recent origin of a novel ADAR recognition motif in *C. elegans* and its closest relatives

**Figure 4:**
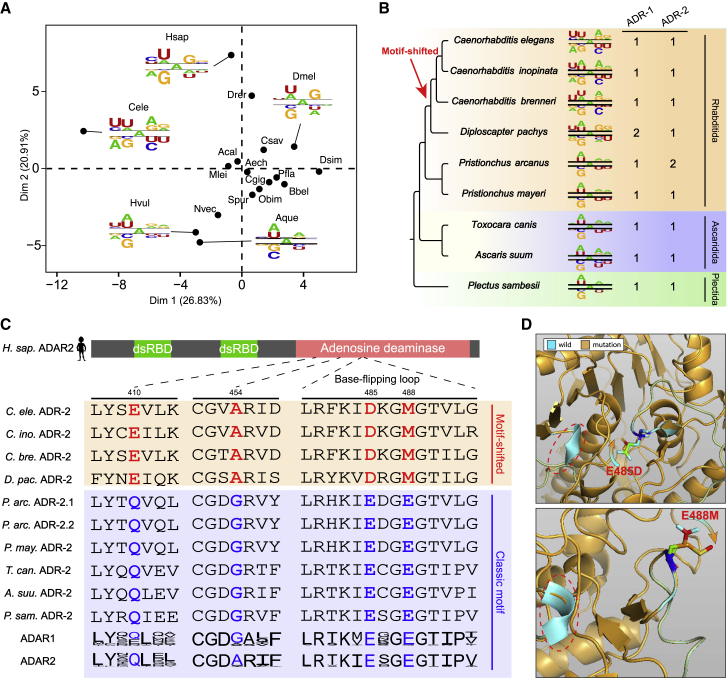
Origin of a novel ADAR recognition motif in nematodes (A) Principal-component analysis based on the neighboring nucleotide preference of the edited adenosines, showing that *C. elegans* is separated from other metazoans based on dimension 1. (B) The neighboring nucleotide preferences of the edited adenosines in nine different nematode species. The copy numbers of ADR-1 and ADR-2 are presented for each species. The red arrow indicates the latest emergence of the *C. elegans* motif in the nematode phylogenetic tree. (C) Multiple sequence alignment showing the four amino acid substitutions that have been fixed in the motif-shifted nematodes after diverging from other nematodes. Of note, the frequencies of amino acids obtained from 15 ADAR1s and 21 ADAR2s from the 16 non-Nematoda metazoans are displayed as sequence logos generated by Weblogo 3. The coordinates of the four indicated amino acids are based on human ADAR2 (UniProt: P78563-2). (D) 3D structure simulation of human ADAR2 with the E485D (top) and E488M substitutions (bottom) relative to the wild-type structure. The structure in cyan represents the wild-type structure with E485 and E488, and the structure in orange represents the structure with D485 or M488. Red circles indicate the areas with structural changes after substitutions. See also Figures S4 and S5 and Table S4.

The ADAR enzymes bind any dsRNA without apparent sequence specificity, but once bound, they edit adenosines with certain 5′ and 3′ neighbors more efficiently than others.^55^ By comparing the surrounding sequence context of edited adenosine sites with that of neighboring unedited adenosine sites (i.e., unedited adenosines with an RNA depth ≥2× and within ±50 nt of the focal edited adenosines), we observed clear neighboring nucleotide preferences in all investigated metazoans (Figures 4A and S4). Specifically, the 5′ nearest neighbor of the edited adenosines strongly favored uridine and adenosine but disfavored guanosine across all metazoans. In contrast, the nucleotide preference for the 3′ nearest neighbor was relatively weaker and less conserved, with guanosine being favored and uridine being disfavored in most species. Overall, our results across diverse metazoans are generally in agreement with the known ADAR recognition motif and support that the 5′ nearest neighbor has more influence on editing than the 3′ nearest neighbor.^55^

Interestingly, while the nucleotide preference for positions other than the 5′ and 3′ nearest neighbors is quite weak or absent in almost all investigated metazoans, the model species *C. elegans* represents a notable exception. The edited adenosines in *C. elegans* also displayed a strong nucleotide preference for the 5′ second nearest neighbor, with uridine being the most favored (Figures 4A and S4). This uncommon pattern has also been identified by another recent study.^56^ To trace the evolutionary origin of this novel motif, we analyzed the RNA editomes of eight additional nematode species, including five from the order Rhabditida to which *C. elegans* belongs, as well as two from the order Ascaridida and one from the order Plectida, which represent two sister clades of Rhabditida.^57^ Of note, as no matching DNA-seq data are available for the RNA-seq data of these additional species, we only identified the hyper-editing sites that are highly clustered (Table S4). A comparison of the nucleotide preference of A-to-I editing sites across these nematodes revealed that the *C. elegans* specific motif is limited to *Caenorhabditis* roundworms and their closest relative *Diploscapter pachys*, indicating that this novel motif appeared quite recently during the speciation of Rhabditida nematodes (Figure 4B).

The relatively recent origin of this novel motif also enabled us to identify the potential amino acid changes in ADARs that may account for motif innovation. All eight additional nematode genomes encode orthologs of *C. elegans adr-1* and *adr-2,* as confirmed by our homology searches (Figures 4B and S5). As ADR-1 is catalytically inactivated due to a lack of amino acid residues that are crucial for the catalytic reaction^58^ (Table S4), we only focused on the changes in ADR-2. By examining ADR-2 amino acid substitutions between the motif-shifted and motif-unshifted nematodes, we identified a total of four substitutions that were fixed in the motif-shifted nematodes after diverging from other nematodes. It is particularly noteworthy that two of these fixed substitutions, E485D and E488M (E, glutamic acid; D, aspartic acid; and M: methionine), are located in the base-flipping loop, a region within the catalytic domain of ADAR enzymes that is important for neighboring nucleotide preferences^59^ (Figure 4C). Furthermore, by investigating the amino acids in these four positions in >30 ADAR proteins from the 16 non-Nematoda metazoans collected in this study, we found that E485 and E488, but not the remaining two sites, were indeed ultra-conserved between the motif-unshifted nematodes and the non-Nematoda metazoans and conserved between ADAR1s and ADAR2s (Figure 4C). Therefore, mutations at these two positions are expected to affect the nature of ADARs. This was supported by the 3D structural simulations, which revealed that the substitution of either of these two positions could cause the disappearance of a β-sheet near the base-flipping loop (Figure 4D).

### Recoding editing independently evolved in different phyla but preferentially targets the neural and cytoskeletal systems in Bilateria

**Figure 5:**
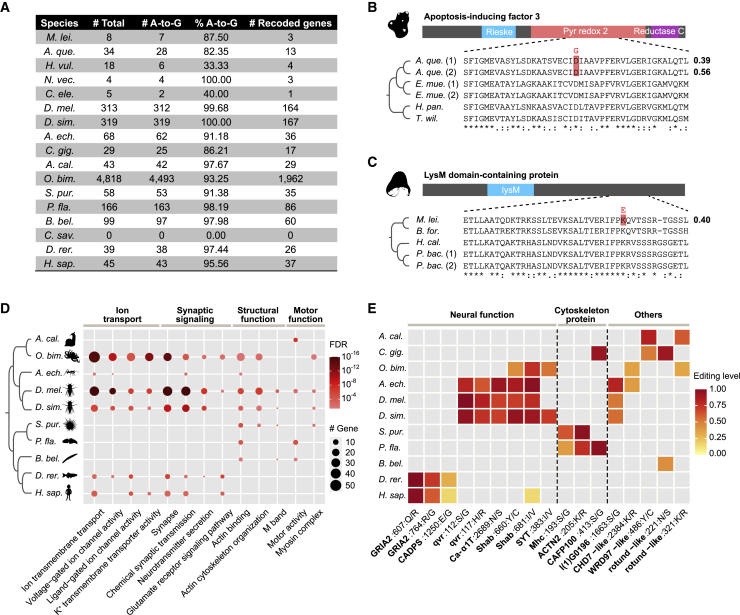
The origin and evolution of recoding editing in metazoans (A) A summary of recoding editing sites identified in each species. (B and C) The recoding of two *AIFM3* genes in the sponge *A. queenslandica* (B) and the *LYSMD3* gene in the ctenophore *M. leidyi* (C). The top part shows the domain organization of the protein products. The bottom part shows the multiple sequence alignments surrounding the recoding sites. The prerecoding amino acids are highlighted by red shadows, and the postrecoding amino acids are shown above the recoding sites. The values on the right side of the multiple sequence alignments represent the editing levels. (D) Functional categories that are enriched by recoded genes in no less than three species (two-sided Fisher’s exact test adjusted p < 0.05). (E) Recoding sites shared by two or more species. For each recoding site, the recoded gene, the protein-based coordinate, the amino acid before recoding, and the amino acid after recoding are shown on the x axis. See also Table S5.

Initially, we found hundreds to thousands of A-to-I editing sites located in coding regions that have the potential to cause nonsynonymous changes in the 17 species with *ADAR*s. However, further examination revealed that most of these putative recoding sites lay within hyper-editing clusters, indicating that they might be the products of the hyper-editing of long dsRNA substrates rather than site-selective recoding editing. On the other hand, a beneficial recoding event is expected to appear across biological replicates and should be edited in notable degree. We thus applied a more stringent framework to search for recoding sites in each species such that these sites must be edited in multiple samples, display an editing level ≥0.1, and appear as an isolated site with few editing sites nearby because functional recoding sites usually result from site-selective editing rather than promiscuous hyper-editing^39^ (see STAR Methods for details). These criteria together greatly reduced the numbers of recoding candidates in all species but well recovered many well-known cases previously identified in vertebrates and insects (Table S5). In addition, the percentages of A-to-G substitution were higher than 80% in most species, demonstrating the high signal-to-noise ratio of these recoding datasets (Figure 5A). Nevertheless, no recoding sites meet our criteria in the sea squirt, and the few sites retained in the hydra and *C. elegans* showed rather low A-to-G signals (33% and 40%, respectively), implying that bona fide recoding sites are likely scarce or absent in these three species. In contrast, the octopus has one to two orders of magnitude more recoding sites than other metazoans (Figure 5A).

Whether recoding editing has evolved in the early branching metazoan lineages remains unknown so far. We identified a total of 31 A-to-I recoding sites that target 16 genes in the sponge and seven recoding sites from three genes in the ctenophore. These included two sponge sites (editing levels: 0.39 and 0.56) that appeared exactly in the same position in two apoptosis-inducing factor 3 (*AIFM3*) genes and caused aspartic acid (polar) to glycine (nonpolar) recoding in the NADH-binding domain (Figure 5B) and one ctenophore site (editing level: 0.40) that caused lysine (basic) to glutamic acid (acidic) recoding in the LysM domain containing 3 (*LYSMD3*) gene (Figure 5C). The prerecoding amino acids in these proteins are highly conserved in other sponge or ctenophore species, implying they are under constraint by natural selection (Figures 5B and 5C). Therefore, the recoding in these conserved positions is expected to be influential in the protein functions. Of note, the number of recoding sites might have been underestimated in sponge/ctenophore, as gene annotations are usually less perfect in these nonmodel species.

To systematically uncover the functional preference of genes subjected to recoding, we next conducted Gene Ontology (GO) enrichment analyses for the recoded genes in each species. In agreement with previous reports, we found that the recoded genes were significantly enriched in ion transport and synaptic signal functions in multiple bilaterian lineages, confirming the important role of A-to-I recoding in modulating neural function^14^^,^^15^ (Figure 5D). But our results also reveal that the preference of recoding neural targets is mostly limited in vertebrates, insects, and cephalopods. The high expression of *ADAR2* in the neural systems of species from these lineages might have contributed to this preference pattern.^7^^,^^8^^,^^33^^,^^60^ In contrast, we found that the cytoskeleton system is likely the more common target of recoding in bilaterians (Figure 5D). Interestingly, besides genes encoding the structural proteins in the cytoskeleton system (e.g., filamin, spectrin, and titin), we also found genes encoding the cytoskeletal motor proteins that convert the chemical energy stored in ATP into mechanical force to be the preferential targets of recoding editing. Representative examples include the dynein axonemal heavy-chain family, which encodes key components of the axonemal dyneins that power the beating of cilia and flagella,^61^ and the muscle myosin heavy-chain family, which encodes the actin-based motor proteins that drive a wide range of motile processes in eukaryotic cells^62^ (Table S5). These results suggest the multiple roles of A-to-I recoding in regulating the metazoan cytoskeleton system.

However, despite the genes involved in neural and cytoskeleton functions frequently appearing as the recoded targets across metazoan lineages, we could only identify one to several recoding sites shared by species from two closely related phyla or shared by species within the same phylum (Figure 5E). This indicates that recoding events were mainly originated independently in each lineage. The possible exceptions are the voltage-gated K^+^ channels (encoded by *shab*), which are well known to display the same recoding events on two highly conserved amino acid residues within the ion transport domain among insects, cephalopods, and even human.^36^

## Discussion

### The origin of A-to-I editing in metazoans

While it has been proposed that the *ADAR* gene family, which encodes the putative adenosine deaminases, originated in the LCA of extant animals,^50^^,^^63^^,^^64^ the bona fide presence and the genomic targets of A-to-I mRNA editing have only been explored in one cnidarian, the coral *Acropora millepora*,^65^ and a handful of bilaterian phyla,^4^ making the origin of this regulatory mechanism in the animal kingdom elusive. Although Porath et al. and Hung et al. recently investigated the landscape of A-to-I mRNA editing across more than 20 metazoan organisms, the species they examined were mostly vertebrates (Chordata) with limited phylogenetic coverage of the animal kingdom.^26^^,^^27^ In addition, the lack of matching DNA- and RNA-seq data for the examined species also prevented the accurate identification of isolated editing sites (e.g., those functionally important recoding sites) in their studies.^26^^,^^27^ By leveraging a matching whole-genome and transcriptome sequencing strategy, we conducted a systematic investigation of RNA editing across the phylogeny of Holozoa for the first time. We identified an overwhelming number of A-to-G substitutions relative to other substitution types in the transcriptomes of metazoans from the earliest branching metazoan phyla (Ctenophora and Porifera) to Chordata but only detected negligible RNA-editing candidates with no substitution-type preference in all the single-celled outgroups. This confirms the long-standing conjecture that the regulatory mechanism of posttranscriptional A-to-I mRNA editing is a metazoan innovation acquired by the ancestor of contemporary animals via the opportunistic gain of *ADAR*s.

The first *ADAR* gene presumably evolved from a tRNA-specific adenosine deaminase progenitor (*ADAT*) through the acquisition of double-stranded RNA binding domains (dsRBDs) that allowed it to work on dsRNA substrates.^49^^,^^66^ Subsequent gene duplication events gave rise to the family members of *ADAR1* and *ADAR2* and the catalytically inactivated *ADAD* observed in extant metazoans.^49^^,^^50^^,^^63^ Our phylogenetic analysis suggests that both *ADAR1* and *ADAR2* could be clearly dated back to the LCA of modern metazoans. The first *ADAD* arose probably in the LCA of modern metazoans as well, and the most parsimonious explanation is in the LCA of bilaterians. This challenges a previous view that *ADAD*s originated after Urochordata-Vertebrata divergence.^50^ In mammals, *ADAR1* is responsible for hyper-editing of repetitive elements in diverse tissues, while *ADAR2* mainly accounts for editing of isolated sites within CDS regions, suggesting a functional divergence between the two *ADAR* members.^7^ However, our broad phylogenetic investigation revealed that species such as the ant *A. echinatior* and the hydra *H. vulgaris* retain only a single *ADAR2* in their genomes but still display high editing activity in repetitive regions. This suggests that *ADAR2* has taken over the hyper-editing role of *ADAR1* in these species. A possible explanation is that both *ADAR1* and *ADAR2* were capable of hyper-editing repetitive elements in stem metazoans, while functional divergence occurred later in some lineages.

### The driving force for the constraint of A-to-I editing in metazoans

Another remarkable feature of metazoan A-to-I mRNA editing is its prevalence across the animal kingdom, as revealed by our data. Secondary loss of the editing machinery is only observed in Placozoa. This raises a question about the primary driving force for the selective constraint of the editing machinery since it originated in stem metazoans. RNA editing is generally viewed as a repair mechanism that corrects harmful DNA mutations at the RNA level in eukaryotes,^2^^,^^3^ such as organelle mRNA editing in land plants^67^^,^^68^ and mitochondrial mRNA editing in kinetoplastid protists.^69^^,^^70^ A textbook example to support this view in animals is that the lethality of mice caused by the failure of Q/R recoding in *GRIA2* could be rescued by replacing the unedited *GRIA2* allele with the edited allele in the genome.^71^ However, it seems to be unlikely that compensation of harmful DNA mutations is the sole driving force, as most editing events in the examined species target repetitive elements that are generally considered to be selfish genetic parasites.^72^ This strong repeat-targeting preference is also a unique feature of ADAR-mediated A-to-I editing that has not been observed for any other RNA editing systems thus far.

Alternatively, recent studies in humans and mice demonstrate that A-to-I editing of endogenous dsRNAs formed by inverted repeats plays a key role in preventing cellular sensing of self dsRNA as nonself (e.g., viral RNA), thus avoiding autoinflammation.^16^^,^^17^^,^^73^^,^^74^ These findings lead to the hypothesis that the main and probably ancestral role of metazoan A-to-I editing is to protect against undesired innate immune responses of endogenous dsRNAs.^4^ Our findings of abundant A-to-I editing in evolutionarily young repetitive elements across a wide range of metazoans support this hypothesis, as the pairing of young repeats with low sequence divergence makes it easier to form long dsRNAs that are also markers of RNA viral infection.^17^ Although further studies are required to confirm the immune response to self dsRNAs in basal animal lineages, a recent study in the planarian *Schmidtea mediterranea* suggested a bilaterian ancestral role of A-to-I editing in suppressing the activation of harmful dsRNA responses.^75^ In addition, it is notable that RIG-I-like receptors (RLRs), which are intracellular dsRNA sensors involved in the ADAR-mediated innate immune response, are also metazoan innovations,^76^^,^^77^ while the placozoan *T. adhaerens* with secondary loss of the editing machinery also lacks RLRs in its genome.^77^ These multiple lines of evidence collectively imply that the emergence of an ADAR-mediated A-to-I editing mechanism was likely a preadaptation that allowed stem metazoans to elaborate their defense mechanisms against RNA viruses by recruiting RLRs into their innate immune systems. In other words, the maintenance of A-to-I editing in extant metazoans might be partly constrained by the existence of certain dsRNA sensors in their genomes.

The evolutionary constraint of metazoan A-to-I editing is also reflected in the common substrate preference (i.e., adenosines with certain neighboring nucleotides in dsRNA regions) across distantly related species. This finding might be of particular value for ADAR-based RNA engineering, such as the recently reported approaches that recruit endogenous ADAR to specific transcripts for site-directed RNA editing in human cells and mice,^78^^,^^79^^,^^80^ as these conserved features imply that the current approaches developed based on mammalian species may well be easily applicable to other metazoan species with *ADARs*.

### The co-option of A-to-I editing into diverse biological processes

As an evolutionary novelty that could introduce single-nucleotide mutations into RNA sequences and that has been firmly established since the LCA of modern metazoans, it is rational to expect that the A-to-I editing mechanism could offer a chance to elaborate the gene regulatory networks during the long-course evolution. This could help explain the versatile characteristics of metazoan A-to-I editing uncovered thus far in model species, including protein recoding, RNA relocalization, the influence of RNA splicing and stability, and the interaction with the RNAi pathway.^4^^,^^5^

Interestingly, in some of the examined metazoans, we found dozens to hundreds of cases with extensive editing of the pairing regions of sense-antisense transcripts. Although once thought to be negligible in mammals,^52^^,^^53^ there is growing evidence suggesting ADAR-mediated RNA editing as a potential way for natural antisense transcripts to regulate the activity of their target genes. For example, in human prostate cancer, the antisense intronic lncRNA *PCA3* inactivates the tumor-suppressor gene *PRUNE2* at the RNA level through an ADAR-mediated mechanism and promotes malignant cell growth.^81^ More recently, antisense RNA-mediated A-to-I editing was demonstrated to play a crucial role in safeguarding against the overactivation of ciliary kinases in *C. elegans*.^82^ Our findings of extensive editing of sense-antisense pairing regions in diverse organisms and the absence of common gene targets across species imply that this might be another mechanism that was exploited independently by metazoans to regulate gene expression. In species such as *C. savignyi*, this mechanism is particularly noteworthy for future investigation given that the majority of A-to-I editing sites in our *C. savignyi* samples indeed came from sense-antisense pairing regions.

The phenomenon of recoding editing has gained additional research interest, as it has the potential to diversify the proteomes or to compensate for harmful DNA mutations.^4^^,^^5^ However, recent studies reveal that most observed recoding events in humans and coleoids are nonadaptive.^83^^,^^84^ Our results extend this generally nonadaptive nature of recoding editing to other animal lineages and support that only a tiny fraction of observed recoding events is beneficial.^83^^,^^85^ It is thus interesting to observe that some recoding sites were shared between distant species at different phyla, suggesting the potential conserved roles of these RNA-editing events. Furthermore, we observe that the cytoskeleton system is likely a common hotspot of recoding editing in bilaterians. The cytoskeleton is an interconnected network of filamentous polymers and regulatory proteins that carries out broad functions, including spatially organizing the contents of the cell, connecting the cell physically and biochemically to the external environment, and generating coordinated forces that enable the cell to move and change shape.^86^ Our findings of both the structural and motor proteins of the cytoskeleton system being frequently recoded thus raise the possibility that ADAR-mediated protein recoding might have been widely but independently exploited to increase cellular complexity during bilaterian diversification.

### Limitations of the study

In this work, we trace the origin and evolution of A-to-I mRNA editing along the phylogeny of Holozoa. While our analyses indicate that A-to-I mRNA editing is a metazoan innovation acquired by the LCA of extant animals via the origin of the *ADAR* gene family, we cannot ascertain which *ADAR* member(s) encodes the catalytically active enzymes in most examined species. The reason is that duplicated genes could undergo functional divergence through processes such as neofunctionalization and subfunctionalization.^87^ That means that the primary role of one or more *ADAR* members might have been shifted from catalyzing A-to-I editing to other functions in a focal lineage during the long course of evolution. In addition, while our findings suggest that ADAR-mediated A-to-I editing might serve as a common regulatory mechanism involved in transposon safeguarding, in antisense-mediated gene regulation, and in protein recoding of neural and cytoskeletal genes in diverse metazoans, following up experimental assays of the RNA-editing sites will be necessary to explore their detail biological functions.

## STAR★Methods

### Key resources table

**Table:** 

REAGENT or RESOURCE	SOURCE	IDENTIFIER
**Biological samples**		

See Table S1 for a complete list of all collected samples	N/A	N/A

**Critical commercial assays**		

TRIzol Reagent	Invitrogen	Cat# 15596026
RNAqueous Total RNA Isolation Kit	Ambion	Cat# AM1912
TruSeq Stranded mRNA LT Sample Prep kit	Illumina	Cat# RS-122-2101
MGIEasy DNA Library Prep Kit V1.1	MGI Tech	Cat# 940-200022-00

**Deposited data**		

Raw sequencing reads	This paper	NCBI: PRJNA557895; CNSA: CNP0000504
RNA-editing sites	This paper	Figshare: https://doi.org/10.6084/m9.figshare.10050437
Refined gene annotations for all investigated species	This paper	Figshare: https://doi.org/10.6084/m9.figshare.10050437
Repeat annotations for all investigated species	This paper	Figshare: https://doi.org/10.6084/m9.figshare.10050437
Original code	This paper	Figshare: https://doi.org/10.6084/m9.figshare.10050437

**Software and algorithms**		

SOAPnuke v1.5.6	(Chen et al.)^88^	https://github.com/BGI-flexlab/SOAPnuke
Pilon v1.21	(Walker et al.)^89^	https://github.com/broadinstitute/pilon
BWA v0.7.15	(Li and Durbin)^90^	https://github.com/lh3/bwa
RES-Scanner v20160713	(Wang et al.)^41^	https://github.com/ZhangLabSZ/RES-Scanner
HISAT2 v2.1.0	(Kim et al.)^91^	https://github.com/DaehwanKimLab/hisat2
Trinity v2.8.4	(Grabherr et al.)^92^	https://github.com/trinityrnaseq/trinityrnaseq
BLAST blast-2.2.26	(Altschul et al.)^93^	https://ftp.ncbi.nlm.nih.gov/blast/executables/blast+/
GeneWise wise2.2.0	(Birney et al.)^94^	https://www.ebi.ac.uk/?birney/wise2/
CDSearch CDD v3.17	(Marchler-Bauer et al.)^95^	https://www.ncbi.nlm.nih.gov/Structure/cdd/wrpsb.cgi
Pfam release-32.0	(Mistry et al.)^96^	https://pfam.xfam.org
RAxML v8.2.4	(Stamatakis)^97^	https://github.com/stamatak/standard-RAxML
Mrbayes v3.2.5	(Ronquist et al.)^98^	https://github.com/NBISweden/MrBayes
PRANK v.170427	(Loytynoja)^99^	http://wasabiapp.org/software/prank/
RepeatMasker v4.0.6	N/A	http://www.repeatmasker.org/RepeatMasker/
RepeatModeler v1.0.8	N/A	http://www.repeatmasker.org/RepeatModeler/
Tandem Repeats Finder v4.07	(Benson)^100^	https://tandem.bu.edu/trf/trf.html
Two Sample Logo v1.21	(Vacic et al.)^101^	http://www.twosamplelogo.org/
DynaMut2	(Rodrigues et al.)^102^	https://github.com/dew111/DynaMut
PyMol	(Schrödinger and DeLano)^103^	https://github.com/schrodinger/pymol-open-source
OrthoFinder v2.2.7	(Emms and Kelly)^104^	https://github.com/davidemms/OrthoFinder
MUSCLE v3.8.31	(Edgar)^105^	https://github.com/rcedgar/muscle
Gblocks v0.91b	(Castresana)^106^	https://github.com/atmaivancevic/Gblocks

### Resource availability

#### Lead contact

Further information and requests for resources and reagents should be directed to and will be fulfilled by the lead contact, Qiye Li (liqiye@genomics.cn).

#### Materials availability

This study did not generate new unique reagents.

### Experimental model and subject details

All the species were either collected from conventionally grown lab conditions, or obtained from the wild. With the exception of the sea hare samples which were purchased from the National Resource for Aplysia, University of Miami, 4600 Rickenbacker Causeway, Miami, FL 33149, samples of all the other species were kindly provided by researchers who have worked on corresponding species for years. The strain identifier (if applicable), geographical origin and providers of each species were listed in Table S1.

### Method details

#### Sample collection

To rule out that false positives resulted from genetic variation during RNA-editing site identification, matching DNA and RNA sequences generated from the same individual/specimen are the ideal data for use in RNA editing studies.^41^^,^^107^ Thus, for the metazoan species with sufficient body mass, both genomic DNA and total RNA were extracted from the same individual, after grinding of the tissue/whole organism in liquid nitrogen. Two to three individuals were collected as biological replicates. These species included the comb jelly *Mnemiopsis leidyi* (three whole adults), the sponge *Amphimedon queenslandica* (three biopsies from three adults), the sea anemone *Nematostella vectensis* (three whole adults), the sea hare *Aplysia californica* (three whole juveniles), the oyster *Crassostrea gigas* (three whole adults after removing shells), the sea urchin *Strongylocentrotus purpuratus* (three pairs of gonad and non-gonad tissues dissected from one female and two male adults; non-gonad tissues comprised the digestive, water vascular, and nervous systems), the acorn worm *Ptychodera flava* (three whole adults), the lancelet *Branchiostoma belcheri* (three whole adults), the sea squirt *Ciona savignyi* (two whole adults) and the zebrafish *Danio rerio* (three whole adults).

For metazoan species from which a single individual is not sufficient to allow the simultaneous extraction of sufficient DNA and RNA for sequencing library construction, 10-15 individuals with similar genetic background were pooled together, then both genomic DNA and total RNA were extracted from the same pool of organisms after the whole pool was ground in liquid nitrogen. These included the hydra *Hydra vulgaris* (10 adults per pool, two pools to serve as biological replicates), the fruit fly *Drosophila melanogaster* (15 male adults per pool, two pools), and *Drosophila simulans* (15 male adults per pool, two pools).

For the unicellular species and tiny metazoan species, biomass was first increased by the propagation of a single colony with the same genetic background, then both genomic DNA and total RNA were extracted from the same culture of organisms. These included the ichthyosporean *Sphaeroforma arctica* (three cultures to serve as biological replicates), the filasterean *Capsaspora owczarzaki* (three cultures), the choanoflagellate *Salpingoeca rosetta* (three cultures) and *Monosiga brevicollis* (three cultures), and the metazoan *Trichoplax adhaerens* (three cultures).

Genomic DNA of all species was extracted with the phenol/chloroform/isopentanol (25:24:1) protocol. The integrity of the DNA samples was assayed by agarose gel electrophoresis (concentration: 1%; voltage: 150 V; Time: 40 min) before DNA-seq library construction. Total RNA of all species except the choanoflagellates was extracted using TRIzol Reagent according to manufacturer’s protocol (Invitrogen, CA, USA). Total RNA of the choanoflagellates *S. rosetta* and *M. brevicollis* was extracted using the RNAqueous Kit (Ambion, CA, USA). The quality of the RNA samples was assayed by the Agilent 2100 Bioanalyzer (Thermo Fisher Scientific, MA, USA) before RNA-seq library construction. In summary, a total of 53 DNA and 53 RNA samples were obtained in this study. After quality control before library construction, two out of the three RNA samples of *M. brevicollis* and one out of the three RNA samples of *N. vectensis* were discarded due to poor RNA integrity (RIN <6).

#### Library construction and sequencing

The strand-specific RNA-seq libraries for all the RNA samples were prepared using the TruSeq Stranded mRNA LT Sample Prep kit (RS-122-2101, Illumina) with 1 μg total RNA as input, then sequenced on the Illumina HiSeq 4000 platform using the PE100 chemistry, according to the manufacturer’s instructions (Illumina, San Diego, CA, USA).

The genomic DNA samples were either sequenced on an Illumina HiSeq 4000 or a BGISEQ-500RS platform. For the Illumina DNA libraries, 1 μg genomic DNA per sample was fragmented by a Covaris ultrasonicator, followed by end repair, 3′-end addition of dATP and adapter ligation. The ligated fragments were then size selected at 300 bp on an agarose gel and amplified by 10 cycles of PCR. The amplified libraries were purified using the AxyPrep Mag PCR Clean-Up Kit (Axygen, MA, USA) and then sequenced on the Illumina HiSeq 4000 platform using the PE100 chemistry according to the manufacturer’s instructions (Illumina, San Diego, CA, USA). The BGISEQ DNA sequencing libraries were prepared using the MGIEasy DNA Library Prep Kit (V1.1, MGI Tech) with 1 μg genomic DNA as input, and sequenced on the BGISEQ-500RS platform using the PE100 chemistry according to the manufacturer’s instructions (MGI Tech Co., Ltd., Shenzhen, China). Details about the sequencing platform and data production for each sample were presented in Table S1.

#### Identification of RNA-editing sites

(i) Quality control for raw sequencing data

All the DNA- and RNA-seq reads were first submitted to SOAPnuke^88^ for quality control by removal of adapter-contaminated reads and low-quality reads before subsequent analyses with parameters *-G -l 20 -q 0.2 -E 60 -5 1 -Q 2*.

(ii) Adjustment of reference genome with DNA-seq data

Given that many samples were collected from wild animals, which have high levels of heterozygosity, or were from strains which are genetically different from those used for assembling the reference genomes, we employed Pilon^89^ to adjust the reference genome of each species using the DNA-seq data from different samples separately, generating sample-specific reference genomes for each species before RNA-editing site identification. Specifically, DNA sequence reads from each sample of a species were first aligned to the published reference genome using BWA-MEM^90^ with default parameters. Then, genome adjustment was performed by Pilon with default parameters except that *--fix snps* was set, using the original reference genome FASTA and the DNA BAM files as input. It is noteworthy that we only adjusted SNPs in the reference genomes in order to ensure that the adjusted genomes from different samples of the same species have the same length and the same coordinate system. The version and source of the original reference genome for each species were listed in Table S1.

(iii) Identification of RNA-editing sites with RES-Scanner

RNA-editing sites from each sample were first identified by RES-Scanner, a software package that was designed to identify genome-wide RNA-editing sites with matching DNA- and RNA-seq data from the same individual or specimen.^41^ Briefly, RES-Scanner invoked BWA-ALN^90^ to align the DNA and RNA reads that passed quality control to the adjusted reference genome of each species, followed by filtering low-quality alignments, calling homozygous genotype from DNA data, and identifying candidate RNA-editing sites from RNA data by ruling out false-positives resulted from genetic variants and sequencing or alignment errors. In general, default parameters were used for the whole pipeline, except that the mapping quality cutoff was set to 5 for DNA alignment (default 20) and the numbers of bases masked at the 5′- and -3′′-end of a DNA read was set to 0 (default 6). This was done as we found that lowering these requirements for the DNA data could yield RNA-editing sites with higher accuracy in many species, manifesting as the higher proportions of A-to-G substitutions out of all identified editing sites.

(iv) Identification of hyper-editing sites

Given that most metazoan A-to-I editing sites tend to occur in clusters, the heavily edited RNA reads (commonly called hyper-edited reads) which contain many of the same type of substitutions in relation to the reference genome, often fail to be aligned during normal alignment process. In order to capture these hyper-edited reads and the clusters of editing sites they harbor, we next performed hyper-editing detection for each sample following a scheme originally proposed by Porath et al.^47^

We first collected the RNA read pairs that could not be aligned to the adjusted reference genome or that had mapping quality <20 from the RNA BAM files generated by the RES-Scanner pipeline as described above. We then removed the read pairs for which one or both reads contained more than 10% of Ns along their lengths, or had particularly large (>60%) or small (<10%) percentage of a single-type nucleotide as recommended by Porath et al.^47^ Next, we adopted a “three-letter” alignment strategy to align these potential hyper-edited reads, in order to overcome the excess mismatches in relation to the reference genome. For example, to align the RNA reads with many A-to-I editing sites (i.e. many A-to-G mismatches), all Ts in the first read of a read pair were transformed to Cs, and all the As in the second read of a read pair were transformed to Gs. This is because, for read pairs generated from the dUTP-based strand-specific RNA-seq libraries, the second read is from the original RNA strand/template while the first read is from the opposite strand.^108^ In the meantime, two versions of the reference genome were created, of which the first version was named the *positive* reference, with all As transformed to Gs, and the second version was named the *negative* reference, with all Ts transformed to Cs.

Next, the transformed read pairs were aligned to both the *positive* and *negative* references by BWA-ALN with parameters *-n 0.02 -o 0*, yielding the *positive* and *negative* alignments, respectively. Then, we filtered both alignments by removing read pairs that were not aligned to the reference genome concordantly, and the reads within concordantly aligned pairs that had mapping score <20. In addition, for *positive* alignment, we further required that the first read in a pair was the reverse complement of the reference genome, while the second read was aligned to reference genome directly; for *negative* alignment, we required that the first read in a read pair was directly aligned to reference genome, while the second read was the reverse complement of the reference genome.

After the strict quality control for the BWA alignments, we converted the transformed reads to their original sequences, followed by trimming the first and last 10 bases of each read in the alignments. Then we identified hyper-edited reads by requiring the mismatch rate of a trimmed read to be >5%, and the proportion of the expected mismatches (i.e. A-to-G substitution in this example) against all mismatches to be >60% as recommended by Porath et al.^47^ Finally, BAM files of hyper-edited RNA reads were submitted to RES-Scanner to extract potential editing sites together with the matching DNA BMA files generated in the previous step. RES-Scanner was run with default parameters in general, except that the mapping quality cutoff was set to 5 for DNA alignment, the numbers of bases masked at the 5′- and -3′-end of a read were set to 0 for both DNA and RNA reads, the minimum number of RNA reads supporting editing was set to 2 (default 3), and the minimum editing level was set to 0 (default 0.05).

The above hyper-editing detection method was undertaken for all of the 12 possible substitution types of RNA editing in each sample of a species, and the results from all the 12 substitution types were combined together by discarding those sites that presented different editing types in any single genomic position.

(v) Combing the results of RES-Scanner and hyper-editing detection

To generate the representative RNA-editing sites for a species, and to improve the identification of editing sites in each sample, we combined the editing sites identified by RES-Scanner (step iii) and hyper-editing detection (step vi) in each sample, to obtain a comprehensive map of potentially editable positions in the reference genome of each species. Specifically, if a genomic position was identified as an editing site in either method, we respectively added the numbers of RNA reads supporting editing, and the number supporting non-editing as generated by these two methods. We then retrieved the missed editing sites in each sample in these editable positions using the criteria of at least one RNA read supporting editing and the false discovery rate (FDR)^109^ adjusted p value for this site to be resulted from sequencing error <0.01. Specifically, statistical tests were performed based on the binomial distribution B(*k*, *n*, *p*), where *p* was set to be the maximal probability of an RNA base to be a sequencing error (i.e. 0.1% here as we only used RNA bases with Phred quality score ≥30), *n* was equal to the total read depth of a given candidate editing site, and *k* denoted the number of reads supporting editing. We also used the DNA-seq data from multiple samples to further remove false-positives resulted from genetic variants, by discarding those editing sites for which the genomic DNA showing the same type of substitution as RNA editing (i.e. the frequency of edited base versus the total number of bases covering this position >0.1) in any one of the multiple DNA samples. RNA-editing sites that displayed different editing types in different samples of a species were also discarded. See Table S1 for the statistics of RNA-editing sites identified in each species.

#### RNA-editing site identification for additional metazoan species

To increase the phylogenetic coverage of the investigated species, we collected the matching DNA-seq and strand-specific RNA-seq data from the nematode *Caenorhabditis elegans* (pooled whole organisms collected from three larval stages and two adult stages),^13^ the leaf-cutting ant *Acromyrmex echinatior* (three pooled head samples of the small worker caste collected from three colonies, respectively),^34^ the octopus *Octopus bimaculoides* (four neural tissue samples including faxial nerve cord, optic lobe, subesophageal ganglia and supraesophageal ganglia)^43^ and human (three brain samples from three male adults, respectively).^10^ The NCBI SRA accession numbers and statistics of the downloaded sequencing data were presented in Table S1. RNA-editing sites in each of the four species were identified using the same procedure (step *i* to *v*) as described above.

#### Refining the ORFs and annotating UTRs for protein-coding genes

Protein-coding genes (GFF/GTF and corresponding cds/pep FASTA files) were downloaded from public databases along with the reference genomes, of which the sources were presented in Table S1. The correctness of the open-reading frames (ORFs) in the GFF/GTF files were checked for all the protein-coding genes, with the defective ORFs such as those that were not the integer multiple of 3 in length or not exactly matching the protein sequences presented in the downloaded pep FASTA files being carefully corrected. Then the transcript model with the longest ORF was chosen as the representative model for a locus if multiple transcript models were annotated in this locus.

5′- and 3′-UTRs for the representative ORFs were annotated using the RNA-seq data used in this study, for all the species except for human. Briefly, RNA-seq reads that passed quality control as described above were first aligned to the reference genome of each species by HISAT2^91^ with default parameters except setting *--rf*, followed by removing those reads that could be mapped to multiple positions of the genome. Then, transcribed regions with continual RNA depth ≥ 5X were extended from the 5′- and 3′-end of each representative ORF to serve as initial 5′- and 3′-UTRs, respectively. Next, an iterative process was used to further recruit the upstream or downstream transcribed regions that were apart from, but linked by ≥ 5 junction reads to previously defined UTRs. If a gene had different 5′- or 3′-UTRs annotated in different samples, the longest one was chosen as the representative 5′- or 3′-UTR for this gene.

#### Analysis of *ADAR* and *ADAD* genes in each species

Protein sequences of *Nematostella vectensis* (GenBank: XP_001642062.2, XP_001629615.2), *Drosophila melanogaster* (GenBank: NP_569940.2), *Caenorhabditis elegans* (GenBank: NP_492153.2, NP_498594.1), *Crassostrea gigas* (GenBank: EKC20855.1, EKC32699.1, XP_011441313.2), *Strongylocentrotus purpuratus* (GenBank: XP_011680614.1, XP_781832.1, XP_030847369.1), *Ciona intestinalis* (GenBank: XP_002128212.1), *Danio rerio* (GenBank: NP_571671.2, NP_571685.2, XP_021334693.1, XP_686426.5, NP_001277142.1, XP_687183.1) and *Homo sapiens* (GenBank: XP_024305442.1, NP_056648.1, NP_061172.1, NP_640336.1, NP_631913.3) collected from NCBI were used as queries to search for *ADAR/ADAD* genes in the public reference genome and the *de novo* transcriptome assemblies (assembled by Trinity^92^) of the 22 species by TBLASTN^93^ with parameters *-F F -e 1e-5*, followed by the determination of protein sequences in the target species with GeneWise.^94^ The predicted proteins were then aligned to the NCBI nr database to confirm whether they were ADARs/ADADs. Domain organizations of the manually confirmed ADAR/ADAD proteins were predicted using the CD-Search tool in NCBI (CDD)^95^ and Pfam^96^ with default settings.

Phylogenetic analysis of ADARs and ADADs identified above, were performed with the adenosine-deaminase (AD) domains (around 324 amino acids in length; see Table S2 for the sequences) using RAxML^97^ with the Maximum Likelihood (ML) method (parameter: *-m PROTGAMMAIJTT*) and using Mrbayes^98^ with Bayesian Inference (BI) method (parameters: *prset aamodelpr = fixed(Wag); lset rates = invgamma; mcmcp ngen = 1000000 nchains = 4 samplefreq = 100 burnin = 200*), respectively. The AD peptide sequences used for phylogenetic analysis were aligned using PRANK.^99^ Reliability of the ML tree was estimated based on 1,000 bootstrap replications. The structures of phylogenetic trees generated by the two methods were generally consistent with each other (Figure S2). The information of *ADAR* genes annotated in each species, including the coding nucleotide sequences, protein sequences, domain annotations are presented in Table S2.

#### Annotation of repetitive elements

Considering that the repetitive elements of many species investigated in this study are either not well annotated and/or not publicly available, we re-annotated the repetitive elements of all the sampled species except human using the same strategy. Repetitive elements of the human genome (GRCh38/hg38) have been well annotated and thus were downloaded from UCSC directly. Repetitive elements in the genomes of the rest species were identified by homology searches against known repeat databases and *de novo* predictions as previously described.^110^ Briefly, we carried out homology searches for known repetitive elements in each genome assembly by screening the Repbase-derived RepeatMasker libraries with RepeatMasker (setting *-nolow -no_is -norna -engine ncbi*) and the transposable element protein database with RepeatProteinMask (an application within the RepeatMasker package; setting *-noLowSimple -pvalue 0.0001 -engine ncbi*). For *de novo* prediction, RepeatModeler was executed on the genome assembly to build a *de novo* repeat library for each species, respectively. Then RepeatMasker was employed to align the genome sequences to the *de novo* library for identifying repetitive elements. We also searched each genome assembly for tandem repeats using Tandem Repeats Finder^100^ with parameters *Match = 2 Mismatch = 7 Delta = 7 PM = 80 PI = 10 Minscore = 50 MaxPeriod = 2000*. To confirm the reliability of our annotations, we compared our repeat annotation results of the fruit fly *Drosophila melanogaster* and the zebrafish *Danio rerio* with those downloaded from UCSC and observed good consistency (Figures S3A and S3B).

#### Identification of clustered editing sites

For each sample of a species, we considered a genomic region containing ≥3 A-to-I editing sites, of which the distance for two adjacent sites was ≤30 nt, as an RNA-editing cluster. The genomic locations of the first and last editing sites in a cluster were assigned as the start and end genomic positions of this cluster. A-to-I editing sites located in the defined editing clusters were regarded as clustered editing sites. To estimate the expected ratio of A-to-I editing sites occurring in clusters in each sample assuming that A-to-I editing randomly occurs in the genome, we randomly selected an adenosine site with comparable RNA depth (i.e., within ±20% of the editing site) for each editing site in a sample, and calculated the ratio of these control adenosine sites occurring in clusters. The significance levels for the difference between the observed and expected ratios were examined by two-tailed paired t-tests in each species (Figure 2B).

#### Estimation of editability for different genomic elements

To compare the editability of different genomic elements, including the protein-coding gene related elements (5′-UTR, CDS, intron and 3′-UTR) and the repeat-associated elements (SINE, LINE, LTR, DNA transposon, Helitron, tandem repeat and other unclassified repeat loci), we calculated the A-to-I editing density for each type of genomic element by counting the number of A-to-I editing sites located in this element type, out of the total number of transcribed adenosines (RNA depth ≥ 2X) from this element type. The editing density of each element type was first calculated for each sample of a species separately, then the mean editing density across samples was calculated as the representative value for a species (Figure 2C).

We also calculated the editing-level-weighted editing densities for each element type (Figure 1, Figure 2, Figure 3, Figure 4, Figure 5S3C and S3D). To do so, an editing site with for example an editing level of 0.1, would be regarded as 0.1 editing site instead of 1 editing site, when counting the number of editing sites for an element type. Only editing sites and transcribed adenosines with RNA depth ≥10X were used in the weighted analysis.

#### Analysis of relationship between repeat divergence and editability

The divergence rates of repetitive elements in each species were estimated by RepeatMasker, by comparing the repeat sequences to the ancestral consensus sequences identified by RepeatModeler during the repeat annotation process as described above. Only the transcribed repeat loci with no less than 50 nucleotides covered by ≥ 2 RNA reads were used for this analysis. The transcribed repeat loci were first sorted according to divergence rate from the lowest to the highest (i.e., the youngest to oldest), then divided into 10 equal bins with the same transcribed repeat loci in each bin. Next the editing density for each bin was calculated, as the number of A-to-I editing sites located in repeat loci belonging to this bin, divided by the total number of transcribed adenosines (RNA depth ≥ 2X) from the repeat loci in this bin. The editing density of each bin was first calculated for each sample of a species separately, then the mean editing density across samples was calculated as the representative value for a species. The relationships between repeat divergence rate and editing density in all species were displayed by a heatmap as presented in Figure 2D.

#### Estimating the potentials of repeat and non-repeat regions to form dsRNA

The potential of repeat and non-repeat genomic regions to form dsRNA was approximatively measured as the ratios of repeat and non-repeat derived genomic sites locating in regions that could find a reverse-complement alignment in nearby regions. Specifically, we randomly selected 100,000 sites from the genomic regions annotated as repeat and non-repeat, respectively. Then, we extracted a 401 nt sequence centered on each randomly selected site and searched this query sequence against a 4001 nt sequence centered on the corresponding repeat or non-repeat genomic site using BLASTN^93^ with parameters *-F F -e 1e-2*. Then a repeat or non-repeat derived genomic site was regarded as locating in a potential dsRNA region formed by intramolecular folding, if a reverse-complement alignment was detected with identity ≥80%, aligned length ≥50 nt, and the aligned region of the query sequence spanned this randomly selected site. The ratio of such sites against all randomly selected sites was calculated to represent the potential of repeat or non-repeat regions to form dsRNA in a species, and the same process was iterated for 100 times to estimate the distribution and significance level (Figure S3F).

#### Identification of editing sites locating in dsRNA regions formed by intermolecular hybridization of sense-antisense transcripts

To identify A-to-I editing sites from dsRNAs potentially formed by the mechanism of intermolecular hybridization of sense-antisense transcripts, we took advantage of the fact that strand-specific RNA-seq preserves the directionality of each RNA reads to examine the RNA read coverage of a 101 nt region centered on each A-to-I editing site, and then searched for editing sites locating in regions with RNA depth ≥ 2X along >50% of the region length on both strands (Figure 3A). As ADARs usually edit adenosines on both strands of the dsRNA substrates, we further searched for editing sites locating in regions (51 nt centered on the focal editing sites) with one or more A-to-I editing sites on the opposite strand (Figure 3B). To estimate the background expectations, we randomly selected a transcribed adenosine site with comparable RNA depth (i.e., within ±20% of the editing site) for each editing site in a sample, and performed the same analyses for these control adenosine sites. The significance levels for the differences between the observed and expected ratios were examined by two-tailed paired t-tests in each species (Figures 3A and 3B).

Given that the observation of A-to-I editing on the pairing regions of both sense and antisense transcripts might happen to be due to an intramolecular folding of the sense and antisense transcripts, respectively, we designed additional filtering steps to completely rule out this possibility. Specifically, for those editing sites locating in regions with A-to-I editing signal on both strands (hereafter referred to as sense-antisense candidates), we extracted a 401 nt sequence centered on each editing site, then searched this query sequence against a 4001 nt sequence centered on corresponding editing site using BLASTN with parameters *-F F -e 1e-2*. Then a sense-antisense candidate was forcedly regarded as locating in a dsRNA region formed by intramolecular folding and discarded, if a reverse-complement alignment was detected with identity ≥80%, the aligned length was ≥20 nt, and the aligned region of the query sequence spanned the edited adenosine. We also removed the sense-antisense candidates if a candidate site resided in an annotated repeat region or in a region with DNA depth exceeding threefold of the peak depth in any DNA sample of a species, as A-to-I editing sites in repeat regions have a higher probability to be resulted from intramolecular pairing of nearby repeat copies. See Table S3 for the statistics and annotation of the high-confidence editing sites in sense-antisense pairing regions.

#### Analysis of the neighboring nucleotide preference for A-to-I editing

The Two Sample Logo software^101^ was used to analyze the neighboring nucleotide preference of A-to-I editing sites with parameters *-K N -T binomial -C nucleo_weblogo -y*. Specifically, for each species, the eleven-nucleotide sequences with the edited adenosines in the center were used as the foreground dataset, while the eleven-nucleotide sequences centered by the transcribed (RNA depth ≥ 2X) but unedited adenosines locating within ±50 nt of the edited adenosines, were used as the background dataset for Two Sample Logo analysis. Nucleotides were plotted using the size of the nucleotide that was proportional to the difference between the foreground and background datasets (Figure S4).

#### Analyses of neighboring nucleotide preference and ADARs in additional nematodes

In order to track the evolutionary origin of the *C. elegans*-specific ADAR motif, we collected the RNA-seq data of eight additional nematode species from NCBI (see Table S4 for accession numbers). As no matching DNA-seq data are available for the RNA-seq data of these additional species, we only conducted hyper-editing site detection with the methods described above. Of note, these RNA-seq datasets were not generated by strand-specific protocols, we therefore regarded both A-to-G and T-to-C substitutions as potential A-to-I editing events. The neighboring nucleotide preference around the hyper-editing sites in each species was analyzed by the method described above. We also performed homology search of ADARs in the reference genomes of these additional species using all the ADAR/ADAD proteins identified in this study (Table S2) as queries and conducted phylogenetic analyses of the nematode ADARs following the methods we described above (see Table S4 for the sequences and domain annotation of the nematode ADARs).

#### Assessing the effects of E485D and E488M substitutions on ADARs

The effects of E485D and E488M substitutions on ADARs were accessed by DynaMut2^102^ on the basis of the human ADAR2 structure (PDB: 5hp2). The wild-type environment was extracted from the 5hp2 A chain. The effects of each of the two focal substitutions were predicted by DynaMut2 following mutation modeling, feature engineering and supervised machine learning. Then the protein 3D structures of wild-type and mutants were visualized in PyMol^103^ (Figure 4D).

#### Identification of putatively beneficial recoding sites

The identification of beneficial recoding sites out of the sea of nonadaptive ADAR byproducts is challenging, as such sites are usually tiny in number, and appear as isolated sites or in small clusters with few editing sites nearby.^39^ To obtain a recoding dataset which is expected to enrich putatively beneficial recoding events, we discarded recoding sites located in hyper-editing regions that contained more than ten editing sites of the same substitution type and the distance for two adjacent sites was ≤20 nt; we discarded recoding sites located in regions (25 nt centered on the focal editing site) that could find one or more editing events of another nucleotide substitutions, as the concurrence of multiple substitution type within a local region is usually indicative of genetic variants or alignment errors. To raise the possibility that the recoding sites were beneficial to the target species, we also required that the recoding sites must be present in two or more samples of a focal species with summed RNA depth ≥10X and average editing level ≥0.1 (see Table S5 for the full list of putatively beneficial recoding sites in each species).

#### Gene ontology annotation and enrichment analysis of recoded genes

GO annotations for the protein-coding genes were downloaded from Ensembl (*Caenorhabditis elegans*, *Ciona savignyi*, *Danio rerio* and *Homo sapiens*) or Ensembl Metazoa (*Mnemiopsis leidyi*, *Amphimedon queenslandica*, *Drosophila melanogaster*, *Drosophila simulans*, *Crassostrea gigas*, *Octopus bimaculoides*, *Nematostella vectensis* and *Strongylocentrotus purpuratus*) via the BioMart function. For *Hydra vulgaris*, *Aplysia californica*, *Acromyrmex echinatior*, *Ptychodera flava* and *Branchiostoma belcheri* that do not have publicly available GO annotations, we first aligned all the proteins of these species to the UniProt database (release-2019_04) using BLASTP^93^ with parameters *-F F -e 1e-5*. Then the best hit of each query gene was retained based on its BLASTP bit score, and the GO annotations of this best hit was assigned to the query gene.

GO enrichment analysis was conducted for genes with at least one putatively beneficial recoding site as defined above. Two-sided Fisher’s exact tests were employed to examine whether the recoded genes of a species was enriched in a specific GO term in relation to background genes, by comparing the number of recoded genes annotated to this GO term, the number of recoded genes not annotated to this GO term, the number of background genes (i.e. the protein-coding genes with RPKM >1 in at least one sample after excluding the recoded genes in the species) annotated to this GO term, and the number of background genes not annotated to this GO term. p-values were adjusted for multiple testing by applying FDR,^109^ and the GO terms with adjusted p-values <0.05 in at least three species (Note: GO terms shared by *D. melanogaster* and *D. simulans* were only counted once here) were considered as the functional categories preferred by recoding editing in metazoans (Figure 5D).

#### Identification of recoding events shared by multiple species

To identify recoding events shared by two or more species (Figure 5E), we first identified the orthologous groups of genes (i.e., gene families) from the seventeen metazoan species with reliable RNA editing using OrthoFinder^104^ with default parameters. For the gene families that contained recoded genes from multiple species, we aligned the protein sequences of the recoded genes using MUSCLE^105^ with parameter *-maxiters 1000* and filtered poorly aligned positions using Gblocks.^106^ Next recoding events occurring in the same position in the alignments and causing the same amino acid changes among at least two species were identified as shared recoding events. Recoding events only shared by *D. melanogaster* and *D. simulans* were removed. Only recoding sites in which the mean editing levels were no less than 0.1 across samples of a species, or were shared by at least two samples, were used in this analysis. The complete list of recoding events shared by multiple species was presented in Table S5.

### Quantification and statistical analysis

All statistical analyses were performed in R. The statistical test used is indicated in figure legends or method details. In Figures 2B, 2E, 3A and 3B, data are presented as mean ± standard error across biological replicates (n = 3 for *M. leidyi*, *A. queenslandica*, *A. californica*, *C. gigas*, *A. echinatior*, *P. flava*, *B. belcheri*, *D. rerio* and *H. sapiens*; n = 2 for *H. vulgaris*, *N. vectensis*, *D. melanogaster*, *D. simulans* and *C. savignyi*), except for *C. elegans* (across five developmental stages), *O. bimaculoides* (across four different neural tissues) and *S. purpuratus* (across three gonads and three non-gonad tissues). Information regarding statistical significance is provided in the figures, with “^∗^” representing p < 0.05, “^∗∗^” p < 0.01 and “^∗∗∗^” p < 0.001.

## Data Availability

• Raw sequencing data generated in this study are deposited in NCBI Sequence Read Archive (SRA) and in the CNGB Nucleotide Sequence Archive (CNSA). Accession numbers are listed in the key resources table. RNA-editing sites, refined gene annotations and repeat annotations generated in this study are deposited in the Figshare repository under the DOI listed in the key resources table. • All original code has been deposited in the Figshare repository under the DOI listed in the key resources table. • Any additional information required to reanalyze the data reported in this paper is available from the lead contact upon request.

## Supplemental information

Document S1. Figures S1–S5

Table S1. The source of biological samples, the summary of data production, the source of genome assembly and gene model annotation, and the statistics of RNA-editing sites identified for each species, related to Figure 1

Table S2. ADAR-like genes identified in the 22 investigated species, related to Figure 1

Table S3. The statistics of A-to-I editing sites located the pairing regions of sense-antisense transcripts, and the sense-antisense pairing regions that were extensively edited on both strands in two or more samples in each species, related to Figure 3

Table S4. ADAR-like genes and RNA-editing sites identified in the eight additional nematode species, related to Figure 4

Table S5. The high-confidence recoding sites identified in each species and the recoding sites shared by two or more species, related to Figure 5

Document S2. Article plus supplemental information
