# Supplementary material for: On the origin and evolution of RNA editing in metazoans
Source: Cell Rep. 2023 Feb 14;42(2):112112. doi: 10.1016/j.celrep.2023.112112 (PMC9989829; doi:10.1016/j.celrep.2023.112112)
Supplement: Document S1. Figures S1–S5 [file mmc1.pdf]

**Cell Reports, Volume *42***

## Supplemental information

# On the origin and evolution

# of RNA editing in metazoans

**Pei Zhang, Yuanzhen Zhu, Qunfei Guo, Ji Li, Xiaoyu Zhan, Hao Yu, Nianxia Xie, Huishuang Tan, Nina Lundholm, Lydia Garcia-Cuetos, Michael D. Martin, Meritxell Antó Subirats, Yi-Hsien Su, Iñaki Ruiz-Trillo, Mark Q. Martindale, Jr-Kai Yu, M. Thomas P. Gilbert, Guojie Zhang, and Qiye Li**

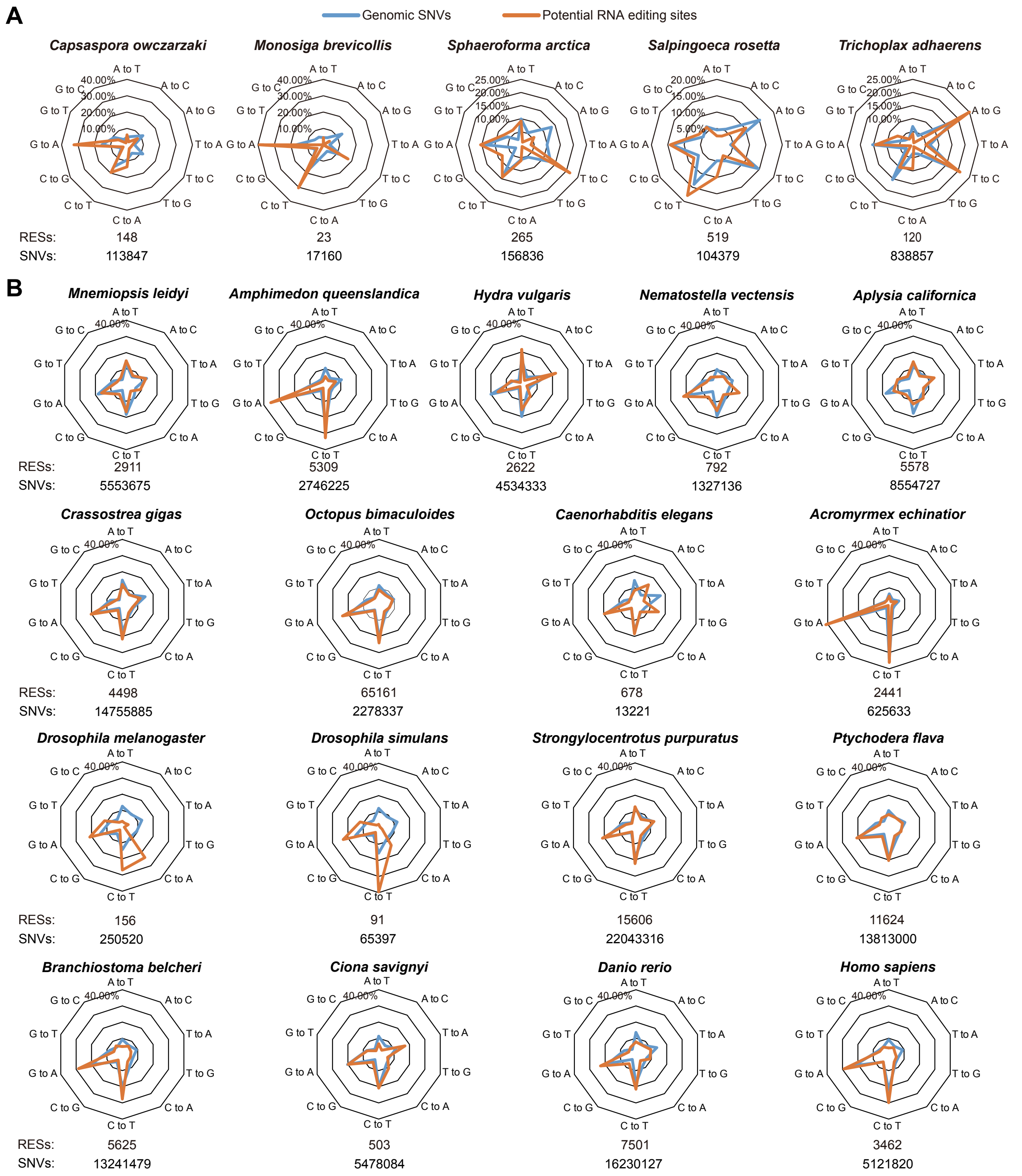


**Figure S1 | The comparison of nucleotide substitution between genomic single nucleotide variants (SNVs) and potential RNA-editing sites (RESs) (related to Figure 1).**

(**A**) The frequency of each type of nucleotide substitution among all SNVs and RESs identified in the five species that lack *ADARs*. Note that the types of nucleotide substitutions for SNVs and RESs were both inferred according to the genotypes present in the plus strand of the reference genome in this analysis. That is, an A-to-G RES from the minus strand of the reference genome was regarded as a T-to-C substitution, while substitution types of RESs from the plus strand remained unchanged. The RESs and SNVs from different samples of the same species were first combined according to their genomic locations, respectively, before the frequency calculation. The numbers of SNVs and RESs are present below each plot. (**B**) The frequency of each type of nucleotide substitution among all SNVs and RESs identified in the 17 species with *ADAR*s, after excluding the substitution types corresponding to A-to-I editing (i.e., A-to-G and T-to-C substitutions inferred from the plus strand of the reference genome).

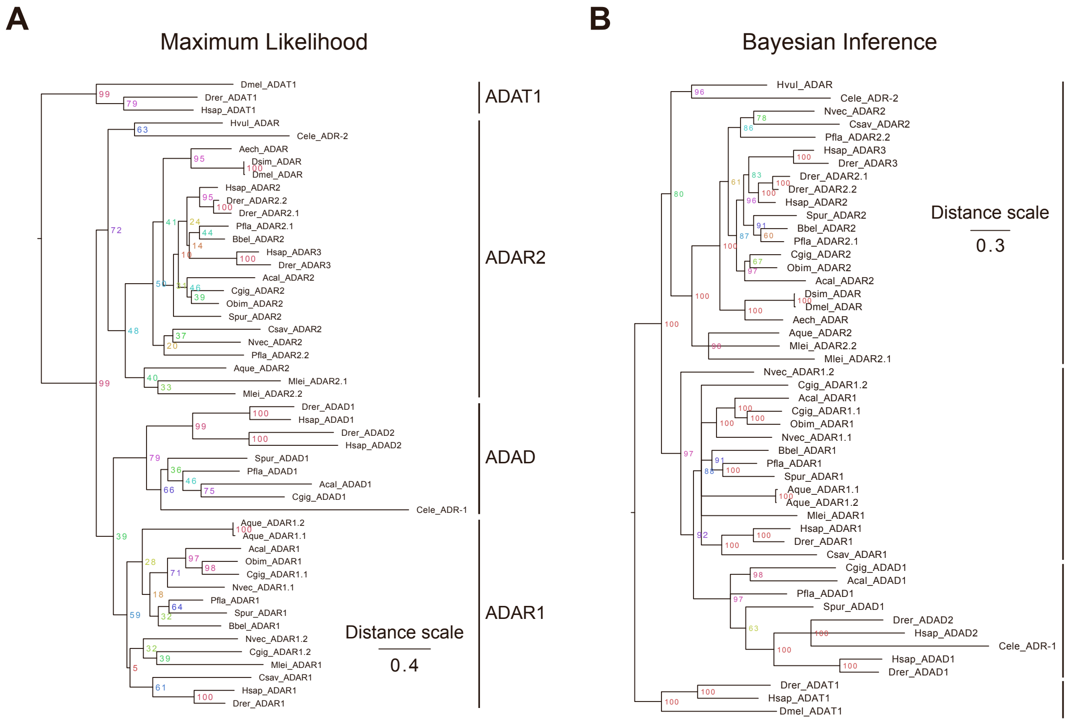


**Figure S2 | The phylogeny of *ADAR/ADAD* genes (related to Figure 1).**

(**A-B**) The phylogenetic trees of ADARs and ADADs based on the peptide sequences of the deamination domains according to maximum likelihood estimation (**A**) and Bayesian inference (**B**). The deamination domains of ADAT1 from *D. melanogaster*, *D. rerio* and *H. sapiens* were selected as the outgroups. See also Table S2.

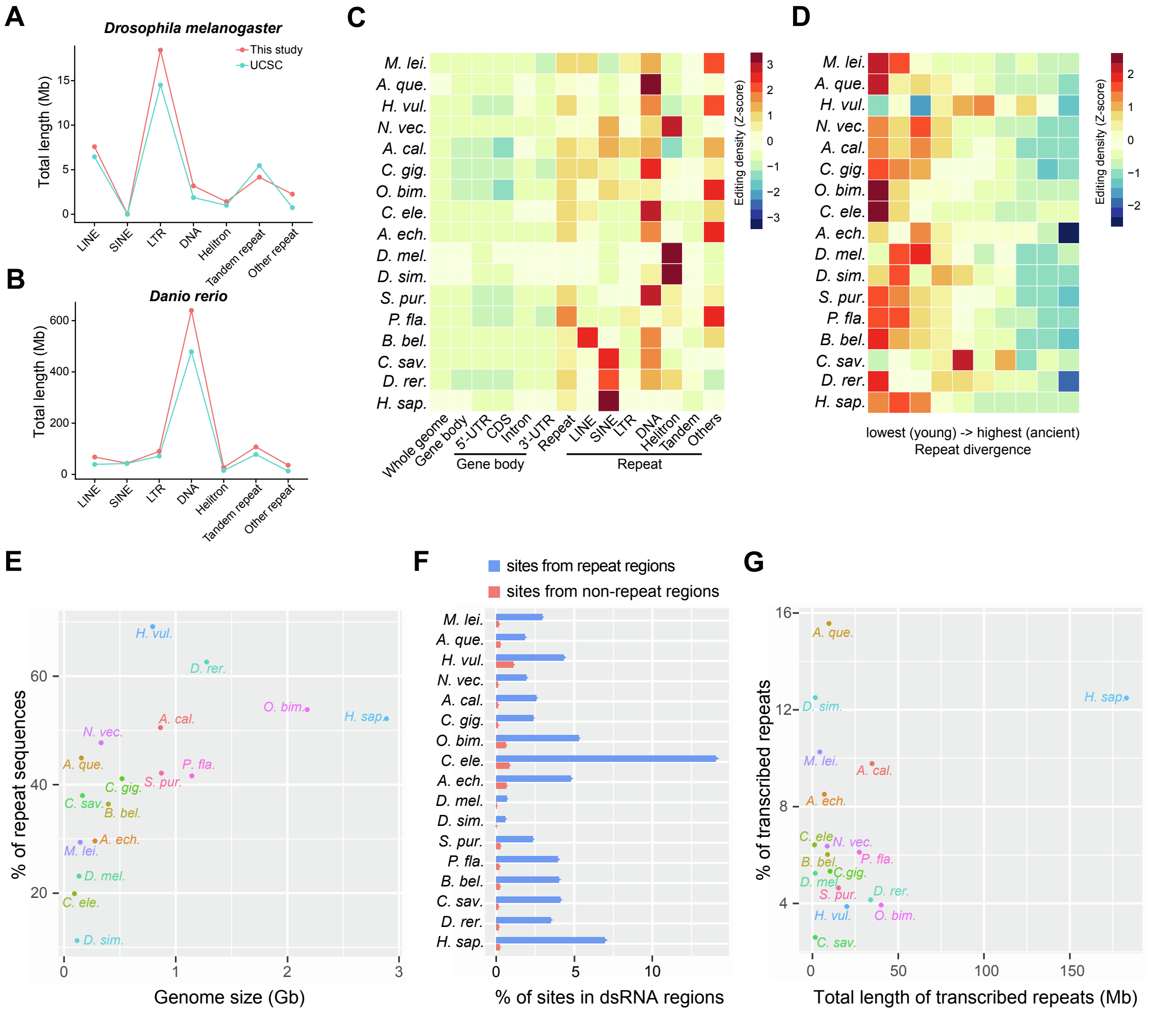

**Figure S3 | The primary genomic targets of metazoan A-to-I editing (related to Figure 2).**
(**A-B**) The non-redundant lengths of different repeat families in *D. melanogaster* (**A**) and *D. rerio* (**B**), according to the annotations generated in this study and those from UCSC, showing the good consistency between these two annotation results. (**C**) Comparison of editing-level-weighted editing density across different genomic elements in each species. The weighted editing density of an element was calculated as the summed editing level of A-to-I editing sites (RNA depth ≥ 10X) locating in this element divided by the number of transcribed adenosines (RNA depth ≥ 10X) in this element. (**D**) The negative correlation between the sequence divergence and editing-level-weighted editing density of repetitive elements. (**E**) Percentage of repeat sequences (summed length of repetitive regions / summed length of non-gap genomic sequences) versus genome size for each species. (**F**) The potentials of repeat and non-repeat regions to form dsRNA in each species, measured as the ratios of repeat and non-repeat derived genomic sites locating in regions that could find a reverse-complement alignment in nearby regions (see Methods). *P*-values were estimated by Monte Carlo simulations (100 times) and < 0.01 for all species. Bars represent the mean ± SD across the 100 times of simulation. (**G**) Percentage of transcribed repeats (summed length of repetitive regions with RNA depth ≥ 2X / summed length of repetitive regions) versus the total length of transcribed repeats for each species. For each species, the sample-specific values were first determined, and then the mean across all samples was presented.

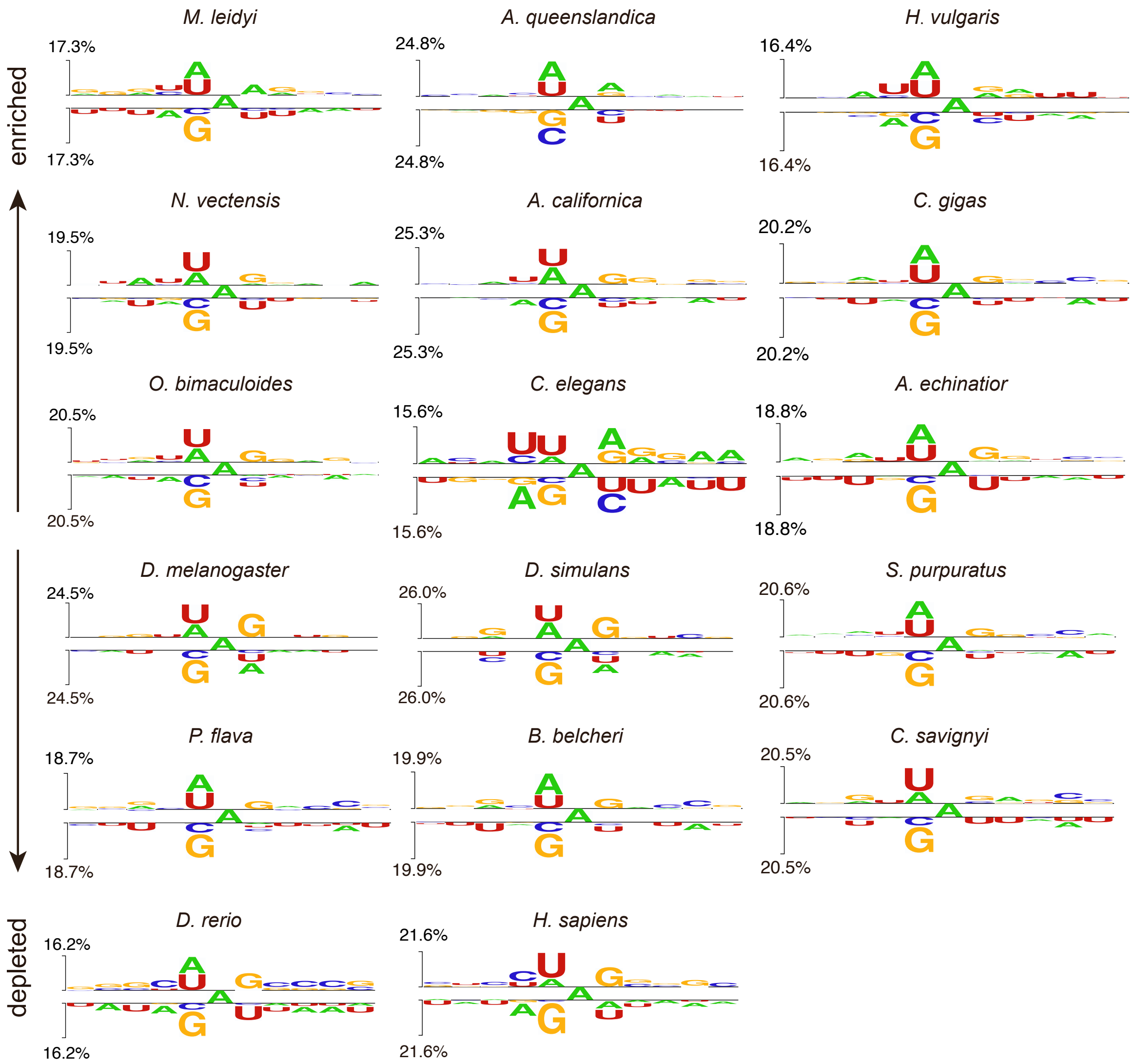


**Figure S4 | Neighboring nucleotide preference of edited adenosines (related to Figure 4).**
The neighboring nucleotide preference (± 5 nt) of the edited adenosines in each species was estimated in comparison to the unedited transcribed adenosines within ± 50 nt of the edited adenosines, by the Two Sample Logo software. Nucleotides were plotted using the size of the nucleotide that was proportional to the difference between the edited and unedited datasets, with the upper part presenting enriched nucleotides in the edited dataset and lower part presenting depleted nucleotides.

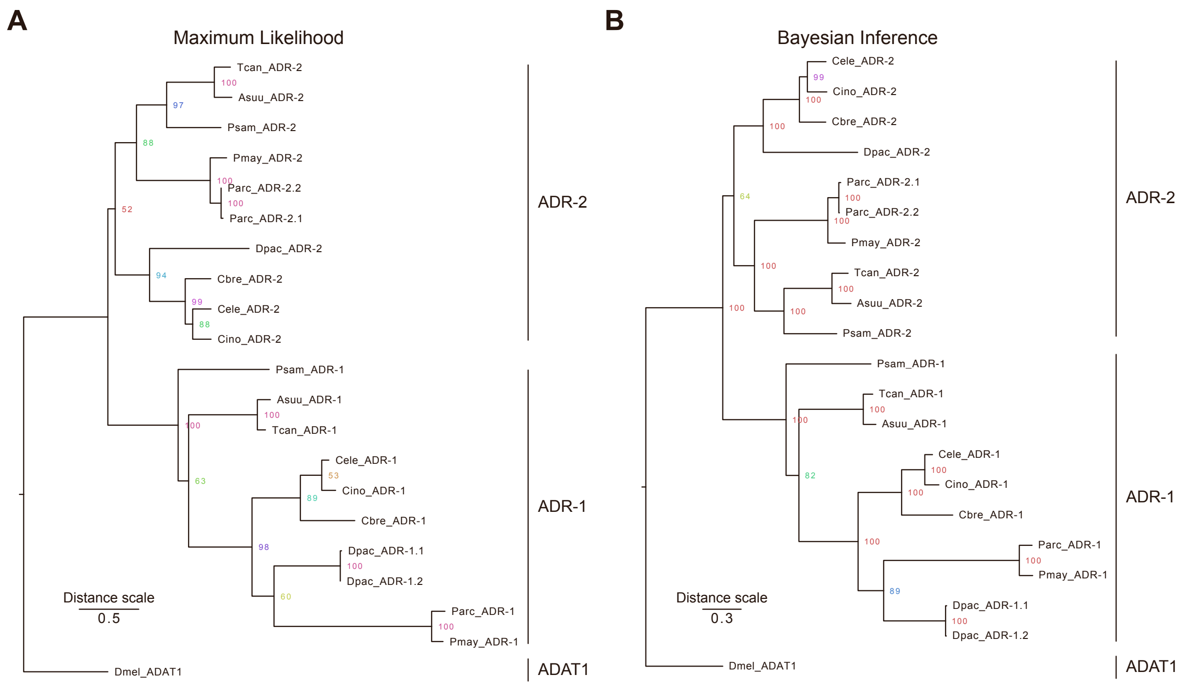


**Figure S5 | The phylogeny of nematode ADARs (related to Figure 4).**
(**A-B**) The phylogenetic trees of ADR-1s and ADR-2s based on the peptide sequences of the deamination domains according to maximum likelihood estimation (**A**) and Bayesian inference (**B**). The deamination domain of *D. melanogaster* ADAT1 was selected as the outgroup to root the trees. See also Table S4.
